# Assembly of functionalized silk together with cells to obtain proliferative 3D cultures integrated in a network of ECM-like microfibers

**DOI:** 10.1038/s41598-019-42541-y

**Published:** 2019-04-18

**Authors:** Ulrika Johansson, Mona Widhe, Nancy Dekki Shalaly, Irene Linares Arregui, Linnea Nilebäck, Christos Panagiotis Tasiopoulos, Carolina Åstrand, Per-Olof Berggren, Christian Gasser, My Hedhammar

**Affiliations:** 1 Division of Protein Technology, School of Biotechnology, KTH Royal Institute of Technology, SE-106 91 Stockholm, Sweden; 2 Department of Solid Mechanics, KTH Royal Institute of Technology, SE-106 91 Stockholm, Sweden; 3 The Rolf Luft Research Center for Diabetes and Endocrinology, Karolinska Institutet, Karolinska University Hospital, S-171 76 Stockholm, Sweden; 4 Linnæus Center of Biomaterials Chemistry, Linnæus University, Kalmar, Sweden; 5 Department of Immunology, Genetics and Pathology, Uppsala University, Uppsala, Sweden

**Keywords:** Biomedical materials, Cell growth, Biological models

## Abstract

Tissues are built of cells integrated in an extracellular matrix (ECM) which provides a three-dimensional (3D) microfiber network with specific sites for cell anchorage. By genetic engineering, motifs from the ECM can be functionally fused to recombinant silk proteins. Such a silk protein, FN-silk, which harbours a motif from fibronectin, has the ability to self-assemble into networks of microfibers under physiological-like conditions. Herein we describe a method by which mammalian cells are added to the silk solution before assembly, and thereby get uniformly integrated between the formed microfibers. In the resulting 3D scaffold, the cells are highly proliferative and spread out more efficiently than when encapsulated in a hydrogel. Elongated cells containing filamentous actin and defined focal adhesion points confirm proper cell attachment to the FN-silk. The cells remain viable in culture for at least 90 days. The method is also scalable to macro-sized 3D cultures. Silk microfibers formed in a bundle with integrated cells are both strong and extendable, with mechanical properties similar to that of artery walls. The described method enables differentiation of stem cells in 3D as well as facile co-culture of several different cell types. We show that inclusion of endothelial cells leads to the formation of vessel-like structures throughout the tissue constructs. Hence, silk-assembly in presence of cells constitutes a viable option for 3D culture of cells integrated in a ECM-like network, with potential as base for engineering of functional tissue.

## Introduction

Since the 1940s, *in vitro* cultures of mammalian cells have become indispensable for both basic research and industrial applications. Most cell culture studies are today performed on hard plastic or glass surfaces because of the ease, convenience and high viability associated with this method. However, forcing cells to adapt against a flat and rigid 2D surface means that almost half of their surface area is dedicated to adhesion, whereas in the body, the cells are likely to receive other signals not just at their ventral surface but in all three dimensions. This can alter the cell metabolism and functionality, thereby providing results different from what would be obtained from cells in their natural *in vivo* environment^1^. Lately, the bearing of culturing cells in 3D has been increasingly acknowledged, and it is expected that 3D cultures provides cellular responses that are of higher biological relevance. When comparing cells cultured in 2D versus 3D, significant differences associated with key biological processes such as adhesion, proliferation, differentiation *etc*. have been found^2–5^.

Promoting cells to grow and form 3D structures *in vitro* has been proven more difficult than first anticipated. By forcing cell-cell contacts to form using *e*.*g*. non-adhesive surfaces and gravity, cells can be gathered into aggregates or spheroids^6^. However, these cell clusters are typically limited in size, since the cells lack a supporting matrix. To permit culture of larger 3D cell arrangements, a temporary support is needed, and for this a range of scaffolds made of both natural and synthetic materials have been developed^7^. Traditionally, a top-down approach has been used, where cells are seeded on top of pre-made scaffolds. Although the scaffolds thereby can be constructed to provide sufficient mechanical support, this approach has limitations in low seeding efficiency and non-uniform cell distribution leading to restricted cell-cell contacts^8^. As alternative, a bottom-up approach has been initiated, relying on assembly from soluble components together with the cells^9^. The choice of material is then limited to those which can be assembled under conditions compatible with cell viability. Several strategies for creation of cellular constructs, *e*.*g*. rapid prototyping and solid free form fabrication, have been developed during the last years^10,11^. One exciting route is 3D printing of hydrogel-based bioinks containing cells^12^. For this, hydrogels which can be induced by mild conditions, *e*.*g*. addition of calcium ions^13^, are used. Thereby the cells are encapsulated in a 3D environment that is beneficial in terms of a high water content. However, from the perspective of a cell, the microenvironment in a pliable reticular hydrogel is very different compared to the native ECM. *In vivo*, the cells are surrounded by networks of ECM containing micro-sized fibers to which they can firmly attach and transmit mechanical forces through^14^. Despite the possibility to fine-tune the bulk mechanical properties of many hydrogels (*e*.*g*. by varying chain length, density and cross-linking), such polymer chains are thinner (nanometer range) than the surface (micrometer range) needed for formation of focal complexes^15^.

We have previously developed a scalable process for recombinant production of the spider silk protein 4RepCT^16,17^. This protein has the unique ability to self-assemble into biodegradable and biocompatible microfibers in aqueous physiological-like buffers at room temperature^17–19^. Further, 4RepCT has been functionalized at the genetic level with a cell adhesion motif from fibronectin (FN) to allow formation of FN-silk, that promotes firm cell attachment^20^. Previous studies of cells seeded on pre-made silk scaffolds showed good proliferation and migration, although cells then grew along the surfaces of the silk rather than inside of the material^20,21^. A proper balance and distribution of cells within the scaffold is crucial for formation of cultures with potential to have tissue-like properties. Our recent discovery about how the air-water interface can be used to promote silk assembly into microfibers^22^ has opened up for a new possibility to efficiently integrate cells into a silk material. Herein we explore the option to include cells already during assembly of FN-silk, with the scope to develop a method for 3D culture of cells integrated in a network of microfibers mimicking the extracellular matrix.

## Silk-Assembly Allows for Direct Integration of Cells Into a 3D Network of Microfibers

In solution, the recombinant silk protein 4RepCT forms dimers of mainly helical/random coil structure^23^. In order to promote a conformational rearrangement driving assembly into a multimeric silk state, alignment against an interface, for example air-water, can be used^22^. The easiest way to formulate silk is thus accomplished by gentle introduction of air bubbles into the solution of silk protein, giving rise to a 3D foam^21^. The silk assembles into a thin film along the walls of each bubble. As the bubbles collapses, these silk films roll up into microfibers, thereby creating a network of randomly distributed microfibers.

**Figure 1:**
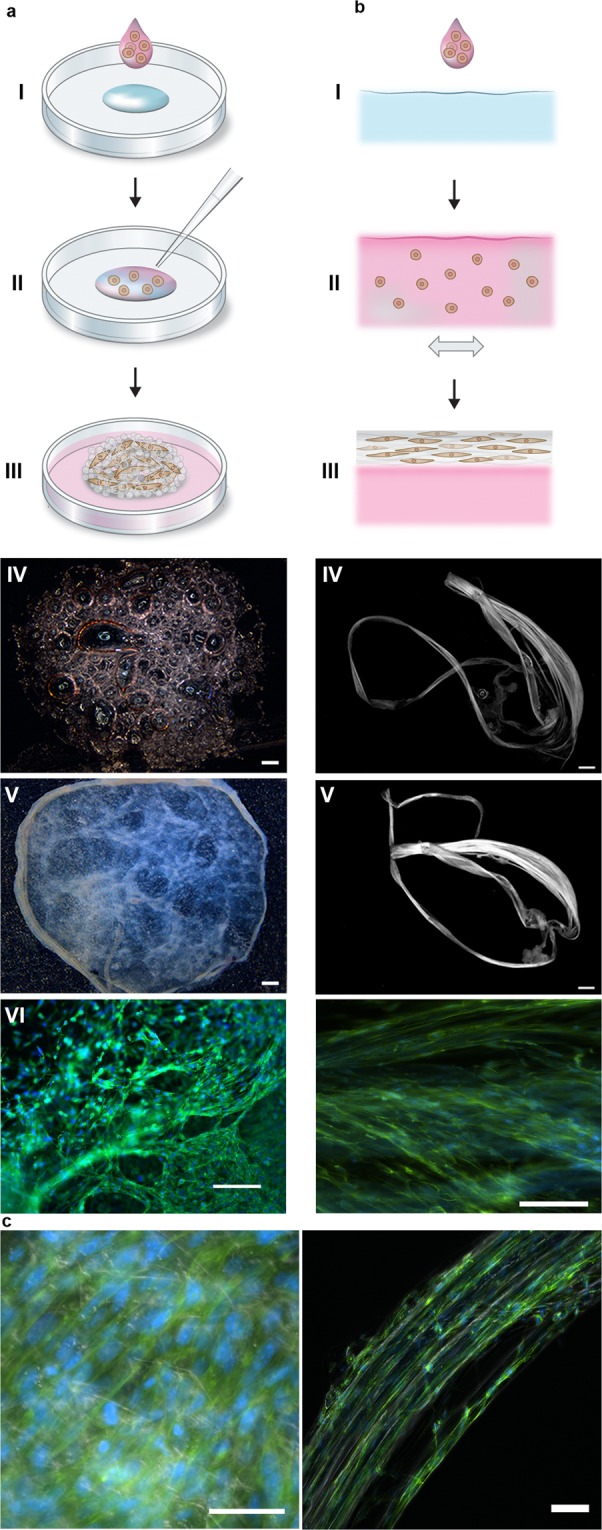
Silk-assembly to integrate cells into a 3D network of microfibers. (**a**) Schematic description of the formulation of silk foam with integrated cells. Cells suspended in culture medium (pink) are added to a defined drop of FN-silk protein solution (blue) placed in the middle of a non-treated culture well (I). Air bubbles are quickly introduced (5–10 sec) through a pipette tip (II), to give rise to a 3D foam with cells. After 30 minutes in the cell incubator, additional culture medium is added to cover the foam completely (III). Day 1 after formulation, the silk foam with cells looks almost transparent, although harboring some remaining air bubbles (disappear with time) (IV). After 2 weeks of culture, the foam with integrated cells shows a denser and whiter appearance (V). Already at day 3 the foam is filled with well-spread cells (here mouse mesenchymal stem cells (MMSC)) (VI). Actin filaments are visualized by phalloidin (green) and cell nuclei by Dapi staining (blue). Scale bar IV-V = 1 mm, VI = 100 µm. (**b**) Schematic description of formulation of silk fibers with integrated cells. Cells suspended in medium (pink) are added to the FN-silk protein solution (blue) (I). During gentle repeated uniaxial tilting for 1–3 hours (II) the silk proteins assemble at the air-liquid interface into a macroscopic bundle of microfibers with incorporated cells (III). The silk fibers with cells are easily retrieved (IV) and can be placed in a well for further culture, whereby the thickness increases over 2 weeks (V). At day 3, aligned cells (here MMSC) are found spread integrated in the fiber bundle (VI). Actin filaments are visualized by phalloidin (green) and cell nuclei by Dapi staining (blue). Scale bar IV-V = 1 mm, VI = 100 µm. (**c**) Differential Interference Contrast (DIC) micrographs of the silk microfibers (white) in a fiber (left) and a foam (right) with integrated cells (MMSC). Actin filaments are visualized by phalloidin (green) and cell nuclei by Dapi staining (blue). Scale bar = 50 µm (left) and 100 µm (right).

To examine the feasibility of this method for formation of a silk network with integrated cells (Fig. 1a,I–III), we first added a drop of dispersed cells (mouse mesenchymal stem cells) to the FN-silk protein solution before assembly. Upon gentle introduction of air bubbles, an expanding wet foam structure was obtained, with similar appearance as a foam made of the silk protein solution by itself. After 30 minutes in the cell incubator, the formed network had settled and remained stable as a cell-containing 3D scaffold even when covered with culture media (Fig. 1a,IV). Throughout the following culture period, the foam became denser and less transparent, as trapped air bubbles were released and the integrated cells increased in number (Fig. 1a,V). Already after 3 days in culture, cells were found spread throughout all dimensions of the foam (Fig. 1a,VI). Using Differential Interference Contrast (DIC) microscopy, a network of silk fibers of micrometer thickness can be seen in the foam with cells (Fig. 1c (left), Suppl. Fig. 1).

The possibility of directing the alignment of cells has been recognized as an essential component of biomimicry^11^. In the foam formulation process, silk-assembly at the walls of the air bubbles results in a random 3D network of microfibers. This network resembles random structures formed in nature, such as the sparsely organized networks of ECM found in loose connective tissue^24^. However, hierarchically ordered structures, with alignment of cells into a certain orientation, is necessary for reconstruction of line-shaped tissues such as blood vessels, nerves, muscle fibers and tendons^8,25^. We have previously shown that a gentle repeated uniaxial tilting of the silk protein solution during the assembly process gives rise to a fiber consisting of aligned silk microfibers that forms a bundle along the air-water interface^16^. Encouraged by the cell compatibility of the foam formulation method, we added cells (mouse mesenchymal stem cells) to the FN-silk solution also before inducing fiber formation by repeated tilting of the interface (3. 1b, I-III). A macroscopic bundle of microfibers started to become visible within the same time frame as seen for silk protein alone (~30 minutes)^26^ and continued to grow the following hours. To the naked eye, the formed fiber with integrated cells first looked similar to those without cells, but continued to grow in thickness during the culture period (Fig. 1b,IV–V). Aligned cells, with prominent actin filaments, were found tightly integrated within the fiber bundle (Fig. 1b,VI). Microfibers of silk can be seen in-between the cells when imaged using DIC microscopy (Fig. 1c (right), Suppl. Fig. 1).

In order to examine the versatility of the silk assembly method, 12 different cell types (see Suppl. Table 1) were investigated for incorporation during the silk formulation process. The studied cell types, mainly isolated human primary cells, were selected to include a wide range of adherent cells, originating from various tissues of the body. The cell to ECM ratio varies widely depending on the tissue^27^ and in some tissues it is of importance that cells are growing densely and in contact with each other. With the silk-assembly method, the added amount of cells, and thus the density, can easily be adjusted to match ranges from the cell sparse cartilage to high-density tissues such as liver^28^. The very simple procedure for formation of silk foam allows for a high cell seeding efficiency, since all the added cells are integrated by the silk assembly process. It is thus a suitable procedure also for 3D culture of cells available only in minute amounts.

## Cells are Highly Proliferative Within 3D Silk

**Figure 2:**
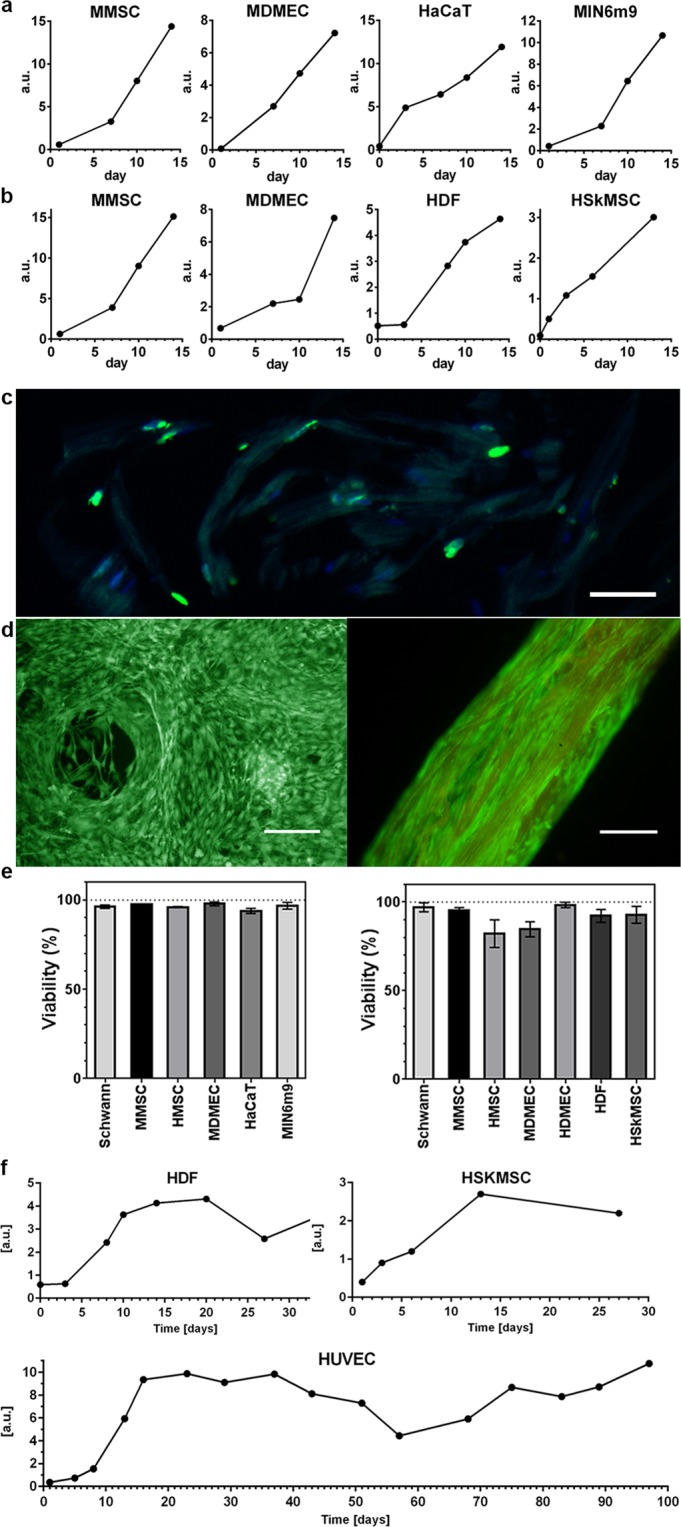
Proliferation and viability of cells integrated in 3D silk. Representative graphs of Alamar blue viability assay show increasing metabolic activity during the first 2 weeks within (**a**) foam (N = 3–4, n = 3–7), and (**b**) fibers (N = 1–9, n = 2–13), reflecting growth of the various integrated cell types (for cell abbreviations see Suppl. Table 1, for details, see Suppl. Table 2). (**c**) Cell division occurs deep within the 3D silk. Cryosection of a fiber with integrated fibroblasts (HDF) fixed at day 11 and stained with FITC-anti BrdU for newly synthesized DNA (green) and Dapi (blue). The silk shows a dim autofluorescence in the blue/green range. (**d**) Representative live (green) and dead (red) staining of mouse mesenchymal stem cells (MMSC) in foam (left) and HDF in fiber (right) at day 14. The fiber shows a dim autofluorescence in the red range. Scale bars = 100 µm. (**e**) Viability (%, mean and standard deviation) after 14 days culture of different cell types (see Suppl. Table 1) in foam (left graph), and in fibers (right graph) (N = 1–3, n = 4). (**f**) Long time cultures of cells integrated into fibers show maintained metabolic activity (Alamar blue) during the entire study period (up to 97 days).

The herein described process for formation of silk scaffolds with integrated cells utilizes silk assembly at the air-water interface, which does not require any additional reagents or conditions known to harm cells (*e*.*g*. changes in pH, temperature, ions and radiation). Still, the entrapment itself might affect the ability of cells to migrate and expand. Therefore, we performed thorough investigations of the growth profiles of the different cells types within both foam and fibers (Fig. 2a,b, Suppl. Fig. 2). In all trials performed, the signal from metabolic activity increased over time, indicating cell proliferation within the 3D silk. Over time, the cell-containing silk scaffolds appeared denser, and microscopy analysis revealed that cells were distributed throughout the scaffolds (Suppl. Fig. 3). To examine if also cells within the innermost part of the silk scaffolds were proliferating, the nucleotide analogue BrdU was added to allow incorporation into the genomes of dividing cells before fixation and cryosectioning. In this way, BrdU positive and thus proliferative cells present deep within the silk fibers could be observed at defined time points (Fig. 2c).

## Cell Viability is High Throughout the 3D Silk

In order to constitute a workable option, it is important that the viability of the integrated cells is maintained during longer culture times. Therefore, a two-color fluorescence assay which simultaneously stains live (green) and dead (red) cells was applied. Under the fluorescence microscope, it became evident that almost all cells were viable after 2 weeks integrated within the silk (Fig. 2d, Suppl. Fig. 4a). The overall viability at day 14, depending on cell type, was 94–98% in foam and 82–98% in fibers, respectively (Fig. 2e). Almost all cells were stained as viable even after longer culture periods (Suppl. Fig. 4b), which could be confirmed by monitoring cultures during 1–3 months using a viability assay (Fig. 2f).

## Comparison of Cell Spreading and Proliferation Within Silk and Alginate Hydrogels

**Figure 3:**
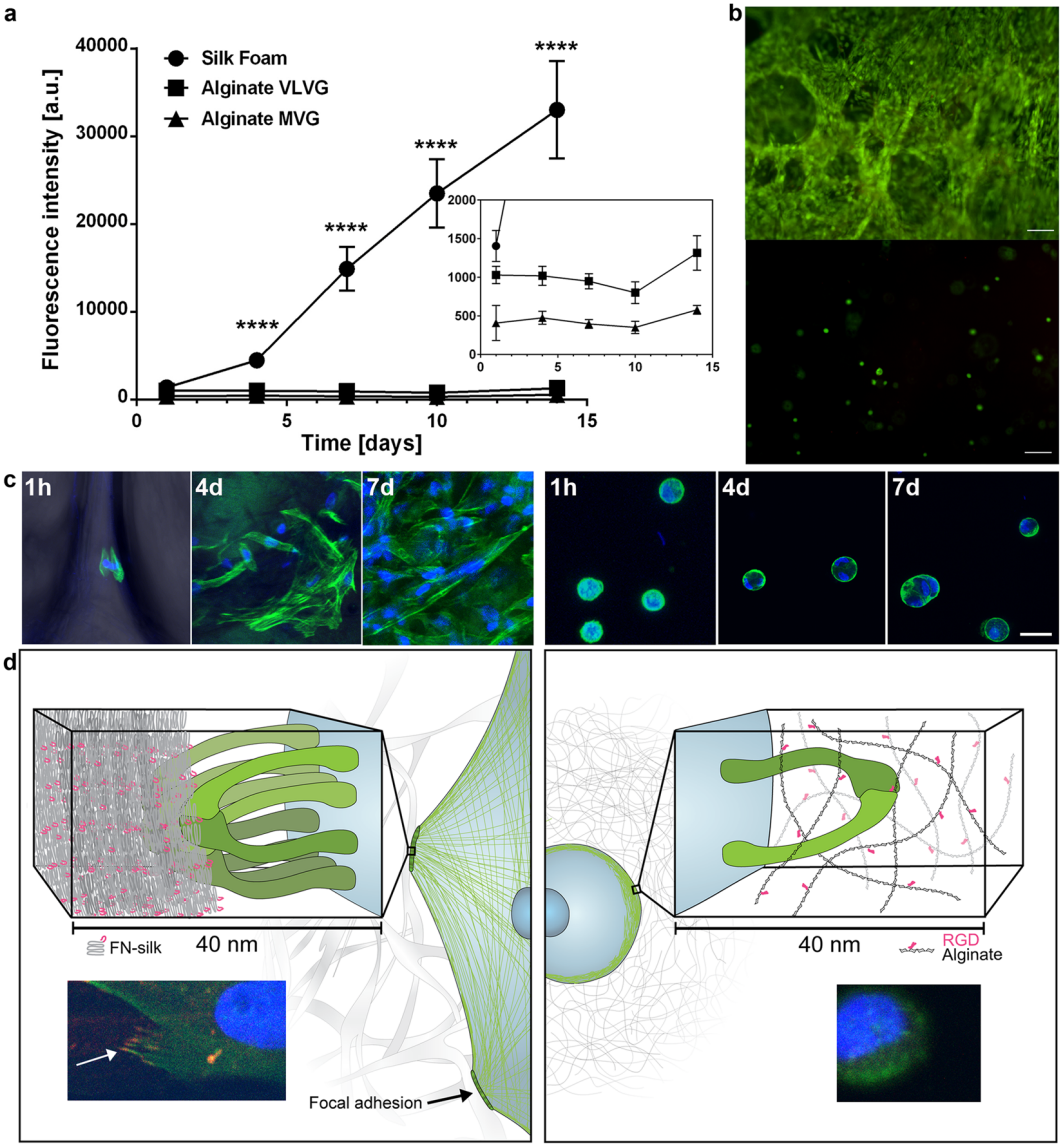
Spreading and expansion of cells within FN-silk compared to when encapsulated in an RGD-coupled hydrogel. (**a**) Representative graph (mean and standard deviation) of Alamar Blue viability assay showing metabolic activity of fibroblasts (HDF) within FN-silk foam (circle), a very low viscosity (VLVG) alginate hydrogel coupled with RGD (square), and a medium viscosity (MVG) alginate hydrogel coupled with RGD (triangle) during two weeks of culture. Insert shows a zoomed in view of the lower intensities. Statistics (students t-test at each time point): **** indicates p < 0.0001 (n = 12) (**b**) Representative live (green) and dead (red) staining of human mesenchymal stem cells (HMSC) in FN-silk foam (upper) and RGD-coupled alginate VLVG (lower) at day 14. Scale bars = 100 µm. (**c**) Confocal scans of HMSCs integrated into FN-silk foam (left panel), and RGD coupled alginate hydrogel MVG (right panel) after 1 h, 4 days and 7 days. Actin filaments are visualized by phalloidin staining (green) and cell nuclei are stained with Dapi (blue). Scale bars = 20 µm. (**d**) Schematic description of our hypothesis for the reason behind the observed difference in interactions between cells and silk (left) versus RGD-coupled alginate hydrogels (right). Several integrin pairs (green) can adhere and gather to the silk microfibers, forming focal adhesions at the edge of actin filaments, enabling the cell to spread and proliferate. In the alginate hydrogel, a single integrin pair (green) can bind to the coupled RGD-motif, but the thin alginate chains restrict subsequent gathering into focal adhesions. Inserts show examples of a cell (fibroblast) after 3 h in FN-silk foam (left) and a low viscosity (VLVG) alginate hydrogel coupled with RGD (right). Actin filaments are visualized by phalloidin staining (green), and focal adhesions can be seen where this is co-localized with staining for vinculin (red, marked with arrow). Cell nuclei are stained with Dapi (blue).

Encouraged by the positive growth profiles obtained from cells integrated into silk, we decided to perform parallel experiments with cells encapsulated in a hydrogel, for comparison. Alginate was chosen as a representative hydrogel since the gelation method is very mild and therefore widely used for cell culture^13^. An alginate variant coupled with the cell binding motif RGD (0.01–0.04 µmole/mg) was chosen to provide cell adhesion sites comparable to the RGD-containing FN-motif. Two versions of G-rich alginate, with different molecular weight and viscosity (MVG, VLVG) were tried. In order to obtain hydrogels with sufficient stability, we chose the commonly used concentration of 2 wt% alginate^29–33^. Still, hydrogels of the low viscosity alginate (VLVG) partly disintegrated during the culture period. When performing parallel experiments with cells in FN-silk and alginate, respectively, the growth curves obtained were markedly different. A clear expansion phase was seen for cells integrated in silk, while cells encapsulated in alginate remained at an almost steady metabolic state (Fig. 3a, Suppl. Fig. 5a). This observation is in line with previous reports of limited proliferation of cells encapsulated in alginate hydrogels^34,35^, although some proliferation has been obtained in compliant hydrogels of 1 wt% alginate^36^. Live/dead staining confirmed a high ratio of viable to dead cells in both scaffold types after 4 hours as well as after 2 weeks of culture (Fig. 3b, Suppl. Fig. 5b).

In order to investigate the reason for the difference in growth behavior, we decided to look closer into the cell microenvironment at selected time points during the culture. Confocal microscopy analysis revealed that cells within silk (both foam and fiber) attained an elongated shape already after 1 hour (Fig. 3c, left, Suppl. Fig. 5c, left), indicating prompt attachment. The cells were increasingly spread out within the silk scaffolds during the culture period, with a clear directional alignment in the fibers (Suppl. Fig. 5c). An increase in cell number during culture in silk scaffolds could also be confirmed from the micrographs (Fig. 3c). When encapsulated within the hydrogel on the other hand, a majority of the cells exhibited a rounded morphology throughout the culture period (Fig. 3c, right). After 4 and 7 days of culture, multiple nuclei could be observed within some of the rounded cell formations, indicating cell division but no spreading when encapsulated in the hydrogel. This is in line with previous reports showing round cells in 2 wt% alginate hydrogels^36^. The authors also report that cells with more spread shape were obtained only within softer (1 wt%) alginate hydrogels which became contracted during culture, suggesting that the cells herein can perceive and react to substrates strains created by the traction stresses of neighboring cells^36^.

Based on the obtained results, we formulated a hypothesis to explain the requirements of the microenvironment to obtain tissue-like culture of cells in 3D (Fig. 3d). In order for cells to attain an accurate cell morphology, they need to assemble several integrins for the formation of focal adhesions in order to trigger organization of the cytoskeleton^37^. Formation of such focal adhesion points is promoted within the silk network, where the sizes of the microfibers recapitulate the micrometer range dimensions of ECM fibers^27^. Using combined staining of cells integrated in silk, we observe punctate vinculin-rich focal adhesions sequestered to the tips of cell protrusions (Fig. 3d, left, Suppl. Fig. 6), confirming integrin-involved binding of cells to the silk. In the hydrogel on the other hand, the coupled RGD-motif is available for integrin binding, but the very thin alginate chains (one saccharide unit thick) do not physically allow gathering of several integrins to the same spot, which prohibits formation of focal adhesion points. The lack of appropriate dimensions of the scaffold where the adhesion motif is presented would thus explain the rounded morphology of cells within the alginate gels observed herein.

## Silk Fibers With Integrated Cells are Highly Extendable

The stiffness of the surrounding provides crucial signals that affect the fate of cells. The mechanical properties of biological tissue are complex, and especially the wide range of stiffness moduli, covering Pa in brain tissue^38^, kPa in vessels^39^, MPa in tendons^40^ and GPa in cortical bone^41^, is challenging to mimic *in vitro*. When compared with the stiffness of cell culture plates (1 GPa), it is not surprising that 2D culture on such substrates alter the properties of most cell types. Recently, it has been shown that tissue is remodulated by cell forces pulling the ECM fibers^27^, showing the importance of flexible fibers within a scaffold to which cells can attach.

**Figure 4:**
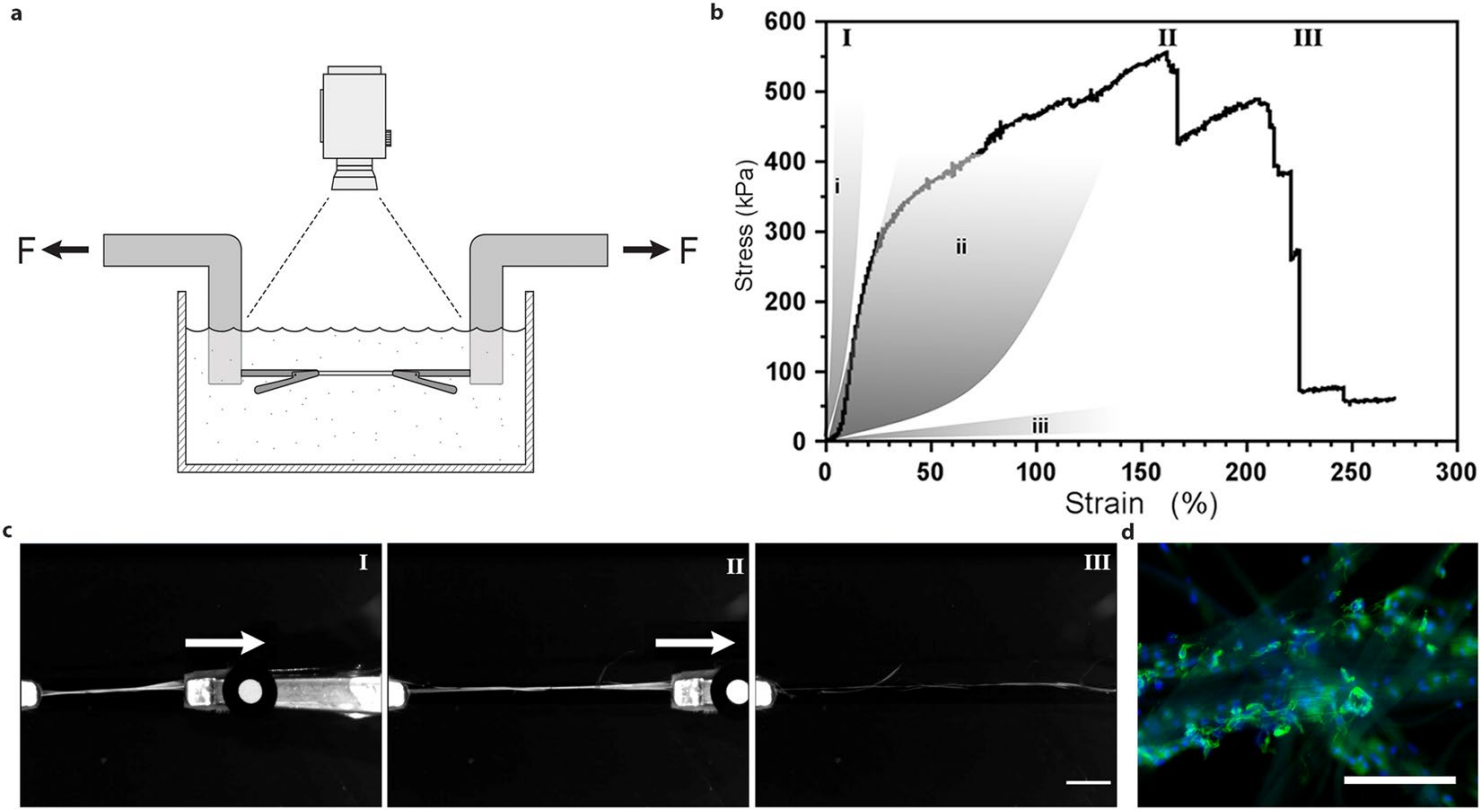
Uni-axial tensile testing of silk fibers with integrated mesenchymal stem cells. (**a**) Illustration of the experimental set-up for tensile tests performed in PBS buffer at 37 °C in order to maintain viable cells. (**b**) Representative First Piola-Kirchhoff stress versus strain curve of a FN-silk fiber with integrated mesenchymal stem cells (MMSC) subjected to tensile testing after 14 days of culture. Stress-strain curve illustrates a rather linear (and probably elastic) phase that is followed by a plastic-like (irreversible) deformation phase until the maximum stress is reached, and the fiber breaks. For comparison, grey areas represent ranges of stress strain properties in tendons and ligaments (i), artery walls (ii), and brain tissue (iii). Roman numbers refer to images (**c**) taken during the tensile test, *i*.*e*. during start (I), extension (II) and breakage (III) of the fibers. Scale bar = 5 mm. (**d**) Micrographs of the breaking point of fibers with MMSCs after tensile testing. Actin filaments are visualized by phalloidin staining (green) and cell nuclei are stained with Dapi (blue). Scale bars = 200 µm.

In order to obtain 3D cellular constructs, it is also important that the scaffold withstands attachment forces, media convections and handling by the personnel. The silk scaffolds with integrated cells constructed herein appeared strong and were stable enough for handling throughout the culture period and analysis procedures. In order to relate the mechanical properties to native tissue, fibers composed of silk microfibers with integrated cells were subjected to tensile testing in a pre-warmed physiological buffer (Fig. 4a). Mesenchymal stem cells, known to attain an elongated cell shape and moderate production of exogenous ECM^42^, were used as a model cell type for these tests. During testing, the fibers exhibited a linear plastic phase (Youngs modulus 1.8 +/− 0.5 MPa) with up to 25% elongation. This was followed by a plastic phase with signs of irreversible deformations (Yield point 338 +/− 107 kPa) and the silk fibers were extended to more than twice its initial length (strain 172 +/− 73%) before breakage (ultimate stress 450 +/− 93 kPa) (Fig. 4b, Suppl. Fig. 7). These results suggest that the mechanical properties of silk with integrated cells match those of connective tissue^43^, with most similarity to artery walls.

Photographs taken during tensile testing confirms that the fibers are highly extendable (Fig. 4c) and show that some microfibers are ruptured before the whole bundle is broken. Micrographs of fiber pieces fixed directly after tensile testing and subsequently stained confirm elongated morphology of the integrated cells (Fig. 4d). Residuals of cells that seem to have their cytoskeleton snapped off can be seen along the ruptured fiber ends. This indicates that a force transition into and throughout the cells has occurred during stretching of the fiber, confirming proper cell attachment.

## Differentiation Capacity of Cells Within 3D Silk

**Figure 5:**
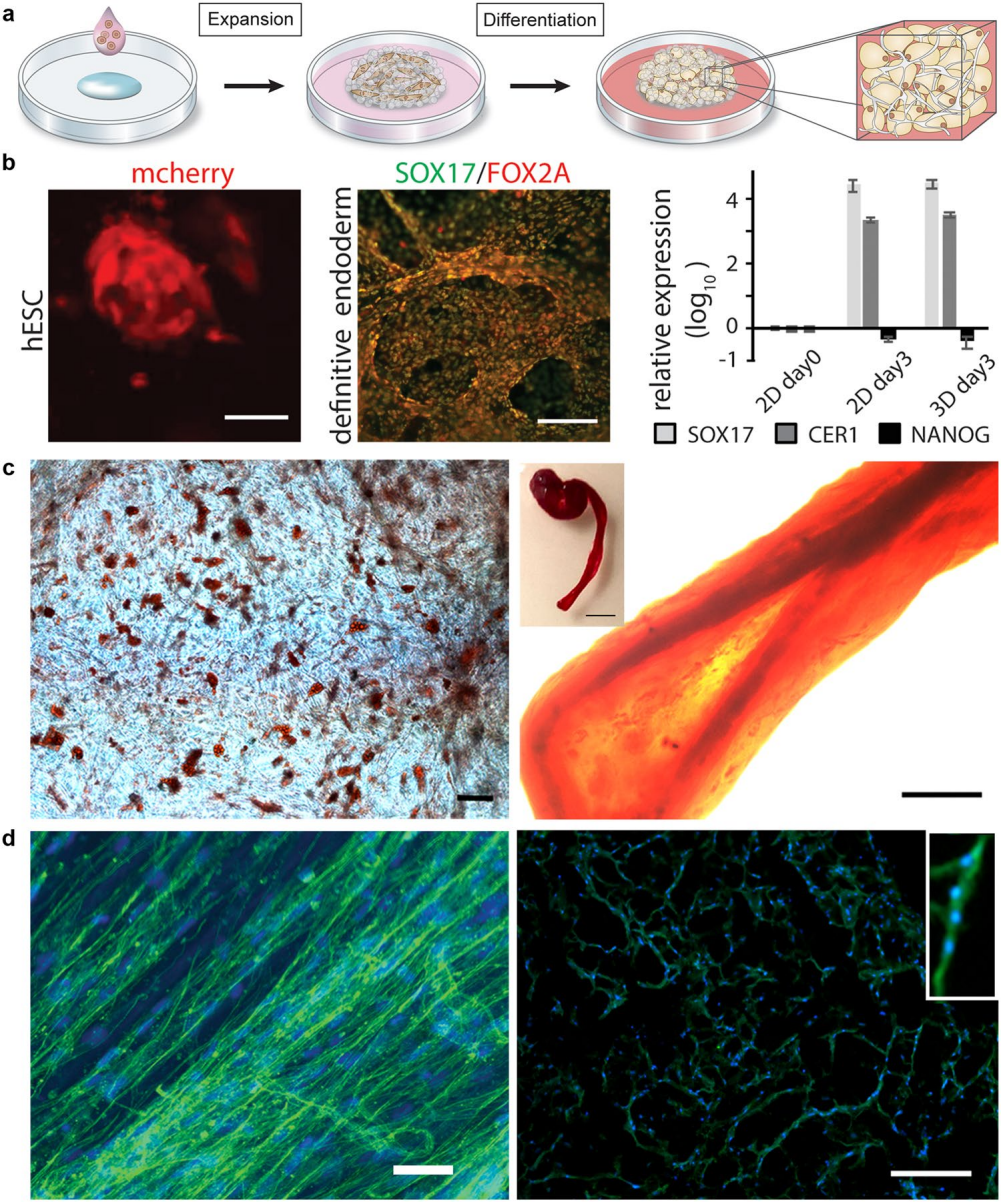
Differentiation of cells in 3D silk. (**a**) After initial expansion of stem cells integrated to 3D silk, differentiation into various tissue types can be triggered by addition of appropriate factors. (**b**) Differentiation of pluripotent stem cells. Left: Human embryonic stem cells (hESC) visualized by mCherry detection at 48 h after cell integration into FN-silk foam. Scale bar = 50 µm. Middle: Immunostaining for endodermal markers SOX17 (green) and FOX2A (red) after 3 days of differentiation. Scale bars = 200 µm. Right: Gene expression (*SOX17*, *CER1*, NANOG) of hESC in a FN-silk foam compared to 2D culture, analyzed by RT-qPCR at day 3 of endodermal induction. Bars represent the mean fold change ± standard deviation (n = 4). (**c**) Differentiation of multipotent adult stem cells. Left: Human mesenchymal stem cells (HMSC) in FN-silk foam differentiated into the adipogenic linage containing lipids, visualized by Red Oil staining (red) (N = 2, n = 4). Scale bar = 100 µm. Right: HMSCs differentiated into the osteogenic linage, probed with osteogenic marker for calcium content (Alizarin Red S (red) in FN-silk fiber (right, scale bar = 200 µm), (N = 2, n = 4). Inset shows photo of a whole fiber (right), scale bar = 1 mm). (**d**) Differentiation of adult precursor cells. Left: After 14 days in differentiation media, skeletal muscle satellite cells (HSkMSC) within a FN-silk fiber show prominent actin filaments, as visualized by phalloidin staining (green). Right: Myogenic differentiation of skeletal muscle satellite cells (HSkMSC) visualized by Desmin staining (green). Dapi-stained nuclei in blue. (N = 9, n = 4). Scale bars = 200 µm. A close up of the area of a multinucleated myotube is shown in the upper right corner.

Recent advances in methods for isolation and expansion of stem cells and the identification of inductive factors that direct differentiation along specific lineages has opened up the potential to provide an unlimited supply of cells for drug testing, research and medical transplantation. In order to engineer complete tissue models from stem cells, it is essential to create realistic artificial stem cell niches. The differentiation niches of stems cells *in vivo* are inherently 3D, and their biochemistry and topology strongly affect the differentiation process^44^. Therefore, we investigated the applicability of the herein described 3D culture set up for efficient differentiation, using both pluripotent and multipotent stem cells (Fig. 5).

Although more sensitive than most adult cell types, human embryonic stem cells could be viably integrated within the silk foam. After 48 hours, expanding cell aggregates were found distributed in 3D throughout the foam (Fig. 5b, left). Endodermal differentiation was then initiated, giving rise to dense layers of cells positive for the endodermal progenitor markers forkhead box protein A2 (FOXA2) and SOX17 (Fig. 5b, middle). Endodermal induction was further verified by mRNA expression analysis, confirming robust upregulation of *SOX17* and *CER1*, and down regulation of pluripotency (NANOG) (Fig. 5b, middle and right).

As a representative of multipotent cells, bone marrow-derived human mesenchymal stem cells (hMSC) were integrated within the 3D silk. After expansion, the cells were subjected to differentiation protocols commonly used to steer the fate into adipogenic and osteogenic lineages, respectively (Fig. 5c). Lipid droplets were then found throughout silk scaffolds with integrated cells that had been treated with adipocyte induction media (Fig. 5c, left, Suppl. Fig. 8). Similarly, calcium phosphate was found deposited throughout cultures treated with osteoblast induction media (Fig. 5c, right, Suppl. Fig. 8), confirming successful differentiation.

In order to investigate the potential of 3D culture in silk for differentiation of adult precursor cells, human skeletal muscle satellite cells (HSkMSC) were expanded within silk fibers. After 2 weeks of further culture in myogenic differentiation media, the fibers were completely filled with aligned cells exhibiting prominent actin filaments (Fig. 5d, left). Elongated cells fused together with multiple nuclei and prominent expression of the muscle-specific marker desmin indicates myotube maturation (Fig. 5d, right).

## Co-culture With Endothelial Cells in 3D Silk Results in Formation of Vessels

**Figure 6:**
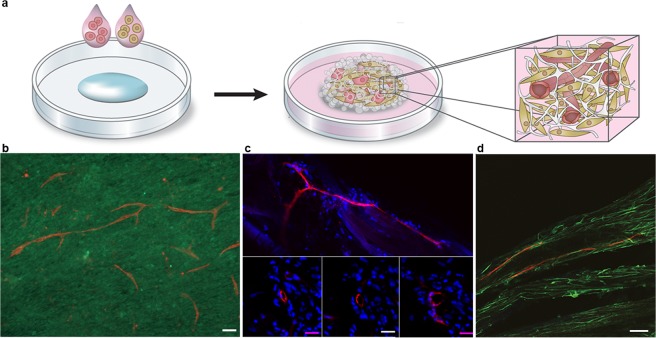
Formation of micro vessels within 3D silk. (**a**) The silk-assembly allows facile combination of two or more cell types. The schematics show an example where addition of a small fraction of endothelial cells together with a connective tissue cell type allows for vascularization of the resulting tissue construct. (**b**) Representative micrograph showing formation of long and branched vessel-like structures in FN-silk foam after 10 days co-culture of 2% endothelial cells (HDMEC, CD31, red) with mesenchymal stem cells (HMSC, CD44, green) in presence of isolated human pancreatic islets (not shown in the image). (N = 5, n = 2). Scale bar = 100 µm. (**c**) Incorporation of a fraction (10%) of endothelial cells (HDMEC) together with skeletal muscle satellite cells (HSkMSC) (upper) or dermal fibroblasts (HDF) (lower) during formation of FN-silk fibers resulted in rearrangement into vessel-like structures. Scale bar = 200 µm. Consecutive cryosections stained for CD31 (red) and nuclei (blue), shows that the vessel-like ring-formation appears at the same position in all three sections (lower). Scale bar = 25 µm (N = 1–8, n = 1–2). (**d**) Aligned CD31 + (red) cells were found in FN-silk fibers with endothelial cells (10%) and skeletal muscle cells after 14 days of culture.

As 3D cultures increases in size, the access to nutrients and oxygen becomes increasingly hampered, since the *in vitro* exchange is based on passive diffusion. In endogenous tissue, this supply is assured through the vasculature network. The lack of vessels thus limits 3D cultures to length scales under which oxygen gradients can occur^45^. The herein described silk assembly method is practically convenient for direct combinations by addition of several cell types to the silk protein solution (Fig. 6a), for example endothelial cells in co-culture with cells from connective tissue. In order to examine the inherent organization capacity for forming microvessels, a fraction of endothelial cells (2–10%) was added together with cells of the connective tissue types before integration by silk assembly (Fig. 6, Suppl. Fig. 9). Already within two weeks, endothelial cells had gathered, and millimeter long branched sprouts were found throughout the co-cultured mesenchymal stem cells in silk (Fig. 6b). Vessel-like structures with prominent rings of endothelial cells were also formed when co-cultured in silk fibers (Fig. 6c). Lumen formations (10–20 µm in diameter) resembling capillaries could be detected at the corresponding location in consecutive cryosections. Various states of vessel formations were also found aligned within the silk fibers after co-culture of endothelial cells and skeletal muscle cells (Fig. 6d).

## Silk Assembly With Cells Allows Creation of Macro-Sized 3D Cultures

The so far presented experiments describe the potential of the silk-assembly method for research purposes. Using silk assembly into 3D foam, a defined amount of recombinantly produced silk protein is used to efficiently integrate all added cells into reproducible 3D cultures in conventional 24-well plates. In order to demonstrate scalability of the methodology to broaden the application range, we will in the following section describe how larger constructs of silk with integrated cells can be achieved.

**Figure 7:**
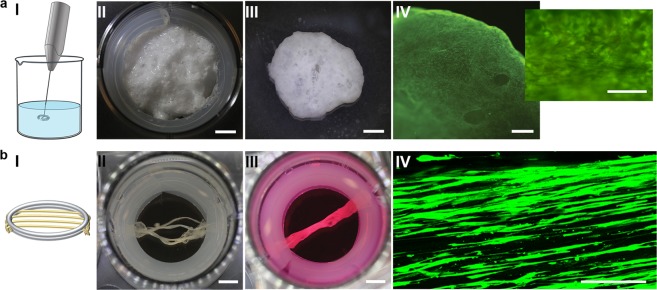
Silk-assembly with cells allows creation of macro-sized tissue constructs. (**a**) Larger foams can be obtained by using a small whisker to introduce air bubbles into the silk within a few seconds (I). A FN-silk foam with integrated HDF placed in a 6-well plate directly after whipping (II). Scale bar = 5 mm. After a few days the foam is free-floating in the well (III). Scale bar = 2.4 mm. Live stain shows a high level of viable cells after 14 days of culture (III). Scale bar = 600 μm. (**b**) Thicker fibers can be obtained by merging several fibers mounted in parallel (I). Several FN-silk fibers with integrated human muscle satellite cells (HSkMSC) were mounted in parallel in a 12 well plate (II), whereby they fuse within 7 days of culture (III). Scale bar = 4 mm. Confocal scan image of the middle plane of a fiber with integrated HDF at day 14 (lower), showing viable and aligned cells (green) inside of the fiber (IV). Scale bar = 50 µm.

In order to obtain bigger foams, we used a small whisker to introduce sufficient amount of tiny air bubbles within a few seconds (Fig. 7a,I). In this case, a suspension of fibroblasts in culture media was added just before whipping. For more sensitive cells, it is also possible to blend the cells with the silk foam directly after whipping. By the use of a spoon, the resulting foam was placed in a culture well fitted with a silicone tube along the edges, to avoid silk attachment to the walls (Fig. 7a,II). After stabilization in the cell incubator for 1 h, the foam was covered with media. Within a few days, the foam detached to end up like a free-floating entity with access to medium from all sides (Fig. 7a,III). After 14 days of culture, viable cells were found throughout the macro-sized foam (Fig. 7a,IV).

The length of the silk fibers obtained is directly dependent on the size of the lab trough used during formation, and thus easily scalable. However, the thickness of the fibers can only reach a certain level, limited by the concentration of protein and cells included. During culture of cells with contractile capacity, such as skeletal muscle cells, we noticed that the fibers curled up if kept free floating, confirming a cooperative action of the aligned cells. Therefore, we investigated the option to mount several fibers in parallel (Fig. 7b,I,II). Within a few days of culture, the fibers with integrated cells had fused to a single millimeter thick fiber (Fig. 7b,III). Confocal microscopy revealed a substantial amount of living cells throughout the depth of the fibers (Fig. 7b,IV).

## Outlook

We present a new strategy for integration of viable cells into 3D networks that mimics the fibrous architecture of native ECM. The mediator for this is the self-assembly of a silk protein that is recombinantly produced, and thereby defined and free from human and animal components. We herein report results showing that the silk formation method to integrate cells into 3D can be applied to a wide range of anchorage-dependent cell types, including endothelial cells, skeletal muscle cells, fibroblasts, keratinocytes as well as pluripotent and multipotent stem cells. The mild and accommodative silk-assembly process occurs simultaneous with cell integration, thereby allowing the cells to migrate and connect to each other. At the same time, a mechanically stable 3D support is provided by the silk. Thereby, intrinsic properties of native tissue, including proliferation in 3D, differentiation capacity and formation of vasculature networks, are reconstituted. Cells gathered within a 3D silk foam constitute a promising option for creation of glandular-like structures, while the fiber shape is more suitable for reconstruction of aligned structures found in blood/lymph vessels, nerves, muscle, ligament, tendon *etc*.

The set-up and protocol for formulation of 3D silk with integrated cells is very simple and can be used by anyone without any need for special instrumentation or technical expertise. Moreover, it is convenient to perform the procedure under sterile conditions, within a laminar flow hood. Compared to time consuming techniques such as 3D-printing, the hands-on requirements for silk-assembly to integrate cells into macroscopic 3D scaffolds are easily fulfilled. The method is simple enough to be widely implemented as standard procedure in any cell lab, rendering 3D almost as easy to obtain as 2D cell culture. Possible applications range from miniature *in vitro* models for drug development to larger transplants for medical treatments.

## Methods

Full details are given in the Supplementary Information.

### Recombinant spider silk protein

The spider silk protein FN-4RepCT kindly provided by Spiber Technologies AB was recombinantly produced in *E*. *coli*, purified using chromatographic methods and subjected to sterile filtration before storage at −20 °C.

### Formulation of silk scaffolds with integrated cells

#### Foam formation

FN-silk protein (3 mg/mL) were placed as a defined drop (15–40 µL) spread out to a diameter of 1 cm in the middle of a well of a non-treated, hydrophobic 24-well plate (Sarstedt). Air was quickly pipetted into the drop 20–30 times. Cells (0.5–2 × 10^6^/mL) suspended in the appropriate culture media (containing 25 mM Hepes, without serum), were added dropwise (10–20 µL) either before or after introduction of air bubbles. The foams were kept 15–60 minutes in the cell incubator before covered with complete cell culture medium. For foams integrated with hESC, 15 µl of FN-silk was used for 50 000 hESCs (15 000 cells/µl). Cells were integrated in the presence of 10 µg/ml Rock inhibitor Y27632 (VWR) and silk was stabilized for 15 min at 37 °C in 5% CO_2_. After stabilization, 0.7 ml NutriStem supplemented with 10 µg/ml Rock inhibitor Y27632 was added. Rock inhibitor was omitted in the medium from 24 hours after seeding. Larger foams were prepared from 1.5 mL of FN-silk (3 mg/mL) subjected to 5–10 sec of whipping using a small battery operated hand whisker (Rubicson).

#### Fiber formation

FN-silk protein (0.5–3 mg) was carefully mixed with cells (0.5–2 × 10^6^) suspended in the appropriate culture medium (containing 25 mM Hepes, without serum) into a total volume of 2–4 mL. Fiber formation was induced by gentle repeated tilting of the lab trough for 1–3 hours at room temperature, while protected from direct light. The formed fibers were washed in pre-warmed 1xPBS and cut into 2–3 cm long pieces. The pieces were placed either mounted across or free-floating in non-treated 12 or 24-well plate with fresh medium during further culture. In order to obtain thicker fibers, several pieces (4–8) were mounted next to each other, enabling fusion during culture.

### Mechanical analysis

Uni-axial tensile test measuring force versus strain (% length extension) of fibers with integrated cells (n = 13) was performed under physiological-like conditions (37 +/− 1 °C, 1xPBS) using a servo-electric custom built Zwick/Roell material testing machine equipped with a 50 N class 1 load cell. The tests were performed on fibers with integrated MMSCs after culture for 14–24 days. Once the 1xPBS buffer bath was heated up, the load cell was leveled and the fiber ends gently mounted in stainless-steel grippers aligned with the machine load line. The tests were performed at a fixed displacement rate of 0.01 mm/s, corresponding to a strain rate of about 0.1%/s. Displacement and load were continuously recorded until the specimen fractured, *i*.*e*. the load dropped. From the recorded tensile load *F*, the First Piola-Kirchhoff stress *P* = *F*/*A* in kPa was calculated, where the fiber’s initial cross-sectional area, *A*, was calculated from fiber dimensions measured with a light microscope (Suppl. Table 3). The strain average (ε) was extracted from the total deformation of the fibers measured at the grip close to the fiber end using video extensometers. The strain *ε* = 100*∙u/L*_*0*_ was calculated by relating the grip displacement u to the fibers initial test length *L*_*0*_. Fibers that broke at the clamps were not considered for the analysis of mechanical properties.

## Supplementary information

Supplementary info
