# Supplementary material for: Assembly of functionalized silk together with cells to obtain proliferative 3D cultures integrated in a network of ECM-like microfibers
Source: Sci Rep. 2019 Apr 18;9:6291. doi: 10.1038/s41598-019-42541-y (PMC6472362; doi:10.1038/s41598-019-42541-y)
Supplement: Supplementary file 1 — Supplementary info [file 41598_2019_42541_MOESM1_ESM.docx]

# Supplementary Information

Assembly of functionalized silk together with cells to obtain proliferative 3D cultures integrated in a network of ECM-like microfibers

Ulrika Johansson^1†^, Mona Widhe^1†^, Nancy Dekki Shalaly^1^, Irene Linares Arregui^2^, Linnea Nilebäck^1^, Christos Panagiotis Tasiopoulos^1^, Carolina Åstrand^1^, Per-Olof Berggren^3^, Christian Gasser^2^, My Hedhammar^1*^

^1^Division of Protein Technology, School of Biotechnology, ^2^ Department of Solid Mechanics, KTH Royal Institute of Technology, SE-106 91 Stockholm, Sweden

^3^The Rolf Luft Research Center for Diabetes and Endocrinology, Karolinska Institutet, Karolinska University Hospital, S-171 76 Stockholm, Sweden

^†^These authors contributed equally to this work

^*^Corresponding author: My Hedhammar ([myh@kth.se](mailto:myh@kth.se))

## Supplementary Methods

1. Cell culture
   1. Primary cell culture
   2. Cell lines
   3. Co-cultures
2. Encapsulation of cells into hydrogels of RGD-alginate
3. Analysis of cells within silk scaffolds and hydrogels
   1. Viability and proliferation assays
   2. Differentiation protocols
   3. Fixation and cryosectioning
   4. Staining and microscopy analysis
   5. RNA isolation and gene expression analysis
4. Statistics

## Supplementary Figures and legends

SF1 Cells integrated into a 3D network of microfibers of silk during self assembly

SF2 Cells are proliferative when integrated within silk scaffolds

SF3 Distribution of cells throughout the 3D silk

SF4 A majority of the cells within 3D silk are viable

SF5 Viability of cells within silk scaffolds compared to an RGD-coupled alginate hydrogel

SF6 Cells within 3D silk form focal adhesions

SF7 Silk fibers with integrated cells are extendable

SF8 Differentiation of stem cells within 3D silk

SF9 Vascularization within 3D silk

## Supplementary Tables

ST1 Cell abbreviations and full names

ST2 Experimental repetitions and parallel replicates

ST3 Dimensions of fibers integrated with mouse mesenchymal stem cells for tensile testing

ST4 Antibody details

# Supplementary Methods

## 1. Cell culture

All cell types used are summarized in Suppl. Table 1.

### *1.1. Primary cell culture*

Human Mesenchymal stem cells (HMSC) (Gibco or PromoCell GmbH, Germany) from bone marrow, were grown in culture flasks coated with CELLstart (Gibco) in complete StemPro MSC serum free medium CTS (Gibco) containing 2mM Glutamax, or in complete MSC Growth Medium DXF (PromoCell), and used at passage 2-8. Mouse Mesenchymal stem cells (MMSC, Gibco) were cultured in DMEM F12 HAM with 10% heat-inactivated Fetal Bovine Serum (FBS) (MSC Qualified, USDA Approved Regions, Gibco) and used at passage 8-14. Human Dermal Microvascular Endothelial cells (HDMEC) (PromoCell) isolated from dermis from adult donors, were cultured in flasks coated with 0.1% gelatin (Sigma Aldrich) in complete endothelial cell media MV2 (PromoCell GmbH, Germany) and used at passage 3-9. Mouse Dermal Microvascular Endothelial cells (MDMEC, Cell Biologics) were cultured in complete endothelial cell medium MV (PromoCell GmbH, Germany) and used at passage 7-9. Human umbilical vein endothelial cells (HUVEC) (PromoCell), were cultured in complete Endothelial cell growth medium (PromoCell), and used at passage 5. Human dermal fibroblasts, HDF (ECACC, Salisbury, UK) were cultured in DMEM F12 HAM with penicillin (100 U mL-1), streptomycin (100 ug mL-1) and 5% FBS (Sigma), and used at passage 8-11. Human skeletal muscle satellite cells, HSkMSC (ScienCell Research Laboratories, Carlsbad, CA) were cultured in Skeletal muscle culture medium, SkMCM (ScienCell), with skeletal muscle cell growth supplement, SkMCGS (ScienCell) and 5% FBS (ScienCell) and used at passage 2-6. Primary human smooth muscle cells isolated from coronary artery, HSMCs (Gibco) were cultured and expanded in complete smooth muscle cell growth medium (Gibco), supplemented with 5% FBS. Schwann cells (ScienCell Research Laboratories) were cultured in Schwann cell medium (ScienCell) with 5% FBS and Schwann cell growth supplement (SCGS, ScienCell) and penicillin/streptomycin solution (3H Biomedical, Uppsala, Sweden) and used at passage 2-6. Medium was changed every 2^nd^ day (HSkMSC and Schwann) or every 2^nd^-3^rd^ days.

### *1.2. Cell lines*

HaCaT (human keratinocyte cell line, spontaneously transformed, CLS Cell Lines Service GmbH), were cultured in DMEM F12 HAM with 5% FBS (Sigma). Medium was changed every 2^nd^ or 3^rd^ day. MIN6m9 (an insulin-secreting mouse pancreatic cell line, RRID:CVCL_4371) was cultured in DMEM (Gibco) with β-mercaptoethanol (50µM), penicillin (100 U mL-1), streptomycin (100 ug mL-1), 10% heat-inactivated FBS and glucose (11 mM) and used at passage 27-35. Medium was changed every 2^nd^ or 3^rd^ day.

Human embryonic stem cell (hESC) line HS975, derived on LN-521 (Rodin et al. 2014) was provided by Karolinska University Hospital, Sweden, and transfected with mCherry. The hESCs were maintained at 37°C, 5% CO_2_ and 5% O_2_ on LN-521 (BioLamina) coated plates in NutriStem (Biological industries) with daily medium change. The cells were passaged with TrypLE (ThermoFisher) and counted manually before integration into silk scaffolds. 15µl of FN-silk was used for 50 000 hESCs (15 000 cells/µl). Cells were integrated in the presence of 10 µg/ml Rock inhibitor Y27632 (VWR) and silk was stabilized for 15 min at 37°C in 5% CO_2_. After stabilization, 0.7 ml NutriStem supplemented with 10 µg/ml Rock inhibitor Y27632 was added. Rock inhibitor was omitted in the medium from 24 hours after seeding.

### *1.3 Co-cultures*

HSkMSC cells in co-culture with 10% HDMEC were cultured in complete SkMCM. HDF cells in co-culture with 3% HDMEC were cultured in complete ECGM MV2. Co-culture of 4000/mL of HMSC and 2000/mL of HDMEC was performed in presence of isolated human pancreatic islets (25 islet equivalents, IEQ) in a mixed culture media containing 50:25:25 of CMRL-1066 (ICN Biomedicals), supplemented with HEPES (10 mM), L-glutamine (2 mM), Gentamycin (50 mg/ml), Fungizone (0.25 mg/ml, Gibco), Ciprofloxacin (20 mg/ml, Bayer Healthcare AG), nicotinamide (10 mM), 10% heat inactivated FBS, StemPro MSC serum free medium CTS (Gibco) with 2 mM Glutamax and endothelial cell media MV (PromoCell GmbH, Germany).

Human islets were obtained from the unavoidable excess of islets generated within the Nordic Network for Clinical Islet Transplantation, from donors agreeing to donate for scientific purposes. Written informed consent was obtained by the National Board of Health and Welfare (Socialstyrelsen), Sweden. Experimental procedures with islets from human donors were done according to the approved ethical permit from the Ethical Committee for Human Research (permit number 2011/1467-32).

## 2. Encapsulation of cells into hydrogels of RGD-alginate

Novatach GRGDSP coupled alginate (Novamatrix, Norway) of high (MVG) or low (VLVG) molecular weight was used to encapsulate cells into 2% hydrogels. Lyophilized VLVG was dissolved in DMEM F12 HAM with penicillin (100 U/mL), streptomycin (100 ug/mL), to a concentration of 4% and then mixed with equal volume of cell suspension at 0.5 x10^6^/mL in the same medium, to achieve a final concentration of 2%. MVG was dissolved directly in the cell suspension (0.25 x10^6^/mL). Gelling was induced by pipetting drops of the alginate/cell suspension (approx. 40 uL/drp, 10,000 cells/gel) into 100 mM CaCl_2_ in medium, incubation at 37 C, 5% CO2 for 1h, followed by 2x wash in PBS/2 mM CaCl_2_ before transfer to appropriate culture media. FN-silk scaffolds were seeded with the same starting number of cells (10,000 cells/scaffold) and cultured in parallel.

## 3. Analysis of cells within silk scaffolds and hydrogels

### *3.1. Viability and proliferation assays*

Alamar blue viability assay (Invitrogen/Life technologies) was used to monitor survival and growth of cells within the scaffolds during culture. The cultures were analyzed every 3-4 days with Alamar blue diluted 1:10 during 1-4 h, depending on cell type. Fluorescence intensity (ex 544 nm, em 595 nm) of supernatants was measured in a multimode plate reader (ClarioStar, LabVision). After withdrawal of blank, each data point was divided by the blank value to correct for inter assay variations, and plotted as arbitrary units (a.u.). In experiments comparing FN-silk and alginate hydrogel, no division with blank was performed. Cultures were continued after addition of fresh medium.

Live/Dead cell viability assay (Molecular Probes) was performed at the selected endpoint, after 7-48 days of culture. The scaffolds were washed in 1xPBS before a mixture of Calcein (1/2000) and EthD-1 (1/500) in complete medium was added to the wells and incubated for 30 minutes in RT. Staining was then analyzed for live (green) and dead (red) cells in a fluorescent inverted microscope (Eclipse, Nikon, Sweden). Using the software NIS-elements 4 equal areas per image was calculated for % viability (amount of green cells/total amount of cells x 100). In addition, confocal scans were taken at 10x magnification at selected plane/-s of the 3D scaffolds for detection of live cells. For proliferation analysis, BrdU (Invitrogen) was added to a final concentration of 10 µM at day 3, 10 and 14 of culture, and incubated for 20 h with BrdU before wash, fixation and cryosectioning. DNA denaturation was performed in 1N HCl on ice for 10 min, 2N HCl at RT for 10 min followed by 20 min at 37°C. Neutralization was done immediately in 0.1M Borate buffer pH 8.5 for 10 min at RT.

### *3.2. Differentiation protocols*

Endodermal induction was initiated two days after integration of hESCs into the FN-silk foam. The cells were cultured for 3 days in RPMI 1640 (ThermoFisher) supplemented with Activin A, 100 ng/ml (R&D), B-27 without insulin (ThermoFisher) and GlutaMAX (Life Technologies). The induction medium was supplemented with CHIR99021, 3 µM (Tocris) during the first 24 hrs.

HMSC integrated into FN-silk fibers or foams were cultured for 7 days before subjected either to adipogenic or to osteogenic differentiation protocols, or used as negative control. HMSC adipogenic and osteogenic differentiation medium (PromoCell) was changed every 3^rd^ day until day 14.

HSkMSC integrated into FN-silk fibers for 14 days were subjected to myotube differentiation protocol, using DMEM medium (Life Technologies, Sweden) supplemented with penicillin-streptomycin solution (100 U mL^-1^, Life Technologies, Sweden) and 2% FBS (Life Technologies) and thereafter changed every second day.

### *3.3. Fixation and cryosectioning*

At selected end points (3h - 97 days), the cell containing scaffolds were fixed in 4% paraformaldehyde for 10-30 min and washed in 1xPBS before storage at +4˚C until stained. Scaffolds to be cryosectioned were incubated in 20% sucrose and embedded in Tissue-Tek (Sakura, Japan), cryo-preserved and sectioned in a Leica CM3050S cryostat to 12-25 µm thick consecutive sections. Sections were dried and stored at room temperature.

### *3.4. Staining and microscopy analysis*

Stainings were either performed on whole scaffolds or on cryosections thereof after permeabilization with 0.1-0.3% Triton X100 in 1xPBS. Blocking was done with 1% bovine serum albumin (Applichem), 5% Normal goat serum, 6% FCS or 10% donkey serum (Jackson ImmunoResearch) in PBS. Primary antibodies: Alexa Fluor 488-conjugated BrdU-mouse monoclonal antibody (Molecular Probes, clone MoBU-1, 4 µg/mL), rabbit-anti-desmin (Prestige antibodies, Atlas antibodies, Sigma, 1:200), mouse anti human vinculin (Sigma, V9131, 9,5 µg/mL), mouse anti human CD31 (BD Pharmingen, 1:100), rabbit-anti-human CD44 (Abcam 1:100), anti-SOX17 (R&D, 1:50) and anti-FOX2A (Abcam, 1:1000). Secondary antibodies: Alexa Fluor 488 goat anti mouse IgG, Alexa Fluor 546 goat anti mouse IgG, Alexa Fluor 488 goat anti rabbit IgG, Alexa Fluor 546 goat anti rabbit IgG, Alexa Fluor 488 goat anti Guinea-pig IgG (all from Molecular Probes, 1:500 or 1:1000), anti-rabbit 647 (Abcam, 1:1000), anti-goat 488 (Jackson ImmunoResearch, 1:1000). Counterstain was done with dapi (1:1000). Slides were mounted in Fluorescence mounting medium (Dako). Micrographs were taken using an inverted fluorescence microscope Nikon Eclipse Ti, or a Leica DMI6000 B. Confocal scans were done using a Carl Zeiss LSM710. Additional stainings used: Phalliodin-Alexa Fluor 488 and Phalloidin-Alexa Fluor 594 (Life Technologies) for f-actin, Hematoxylin-Eosin for nuclei and cytoplasm, the lipid marker Red Oil O (Sigma Aldrich) for fat, and the osteogenic marker Alizarin Red S (Sigma Aldrich) for bone, all according to standard protocols. Antibody identity details are compiled in Suppl. table 4.

### *3.5. RNA isolation and gene expression analysis*

Extraction of total RNA was carried out using a miniRNA purification kit (Qiagen). The RNA was reverse-transcribed using Maxima first strand cDNA synthesis kit (ThermoFisher). Real-time qPCR was carried out with TaqMan probe-based detection in a C1000™ Thermal Cycler (Bio-Rad) according to the manufacturer’s instructions. TaqMan gene expression assays (Life Technologies) were used for: human nanog ID Hs04260366_g1, human B2M ID4333766, human SOX17 Hs00751752_s1, human CER1 Hs00193796_m1. B2M was used as an endogenous control and expression was quantified by 2-ΔΔCt. Results from real time qPCR measurements were expressed as mean fold change ± standard deviation (n=4) or otherwise stated. Data are presented as mean ± standard deviations.

## 4. Statistics

Unpaired Students t-test was used to compare cell viability (by Alamar Blue) in silk foam vs. alginate hydrogel for each time point. *P*-values<0.05 were considered significant.

**Supplementary figures**

a


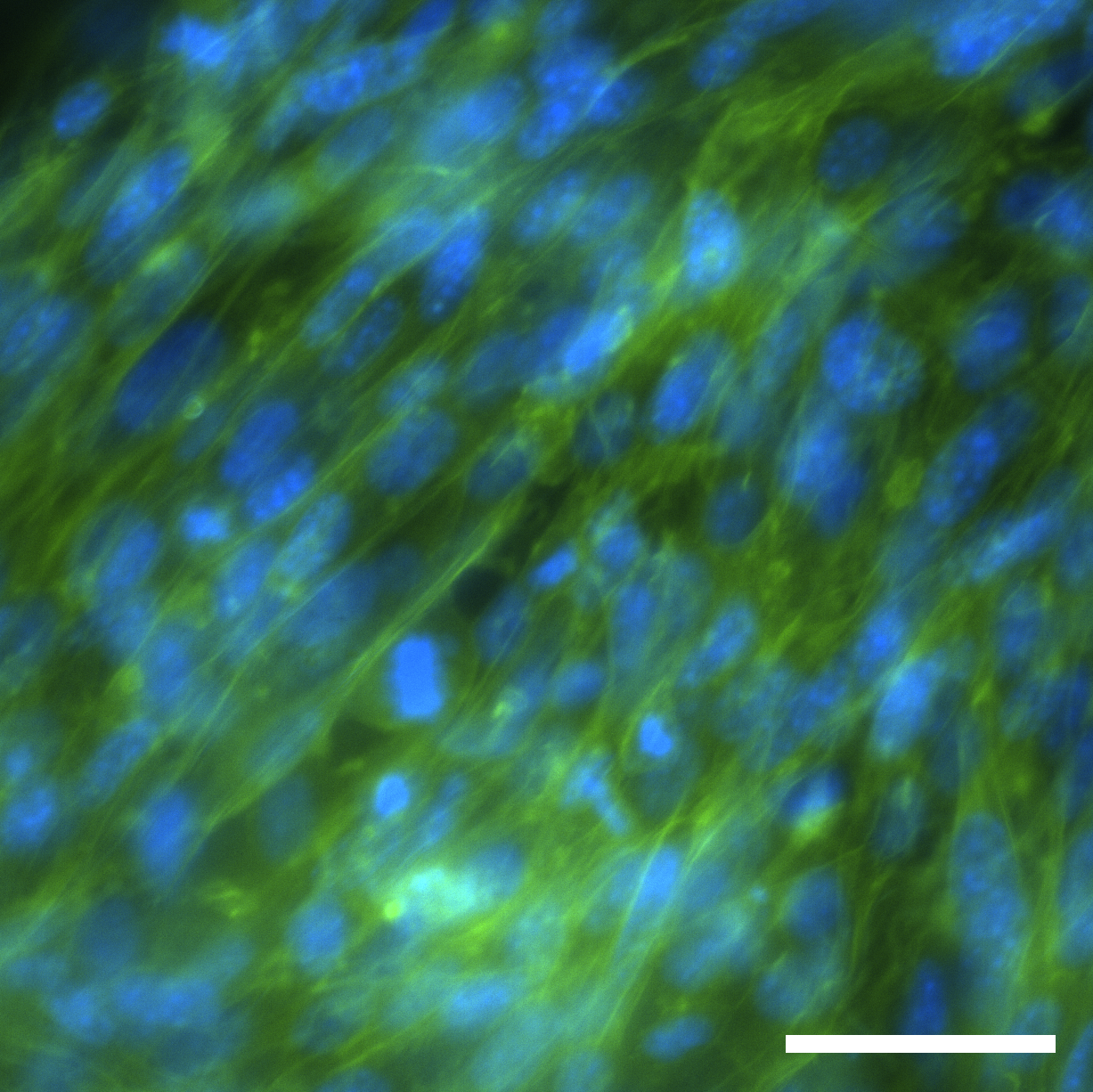

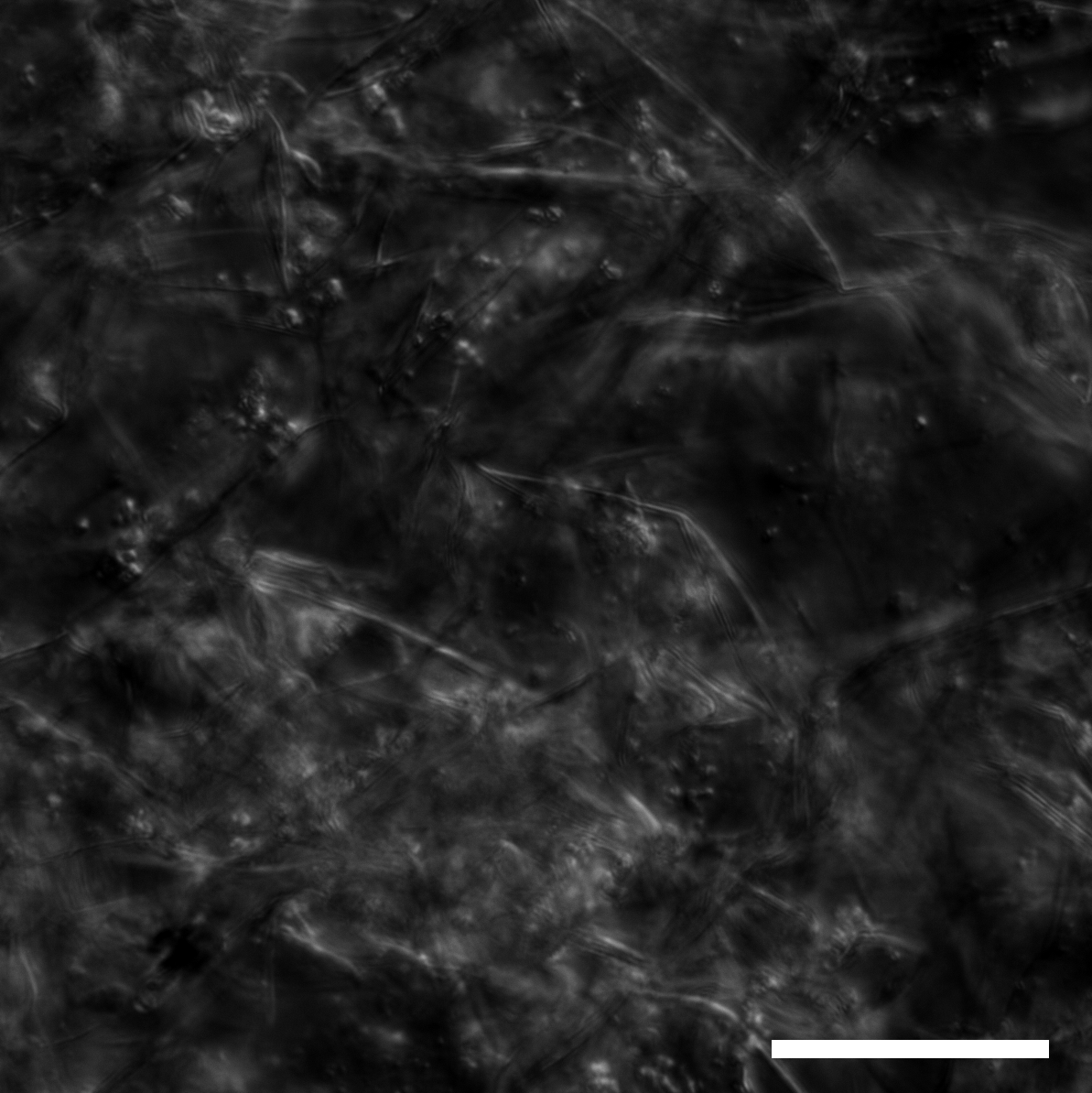


b


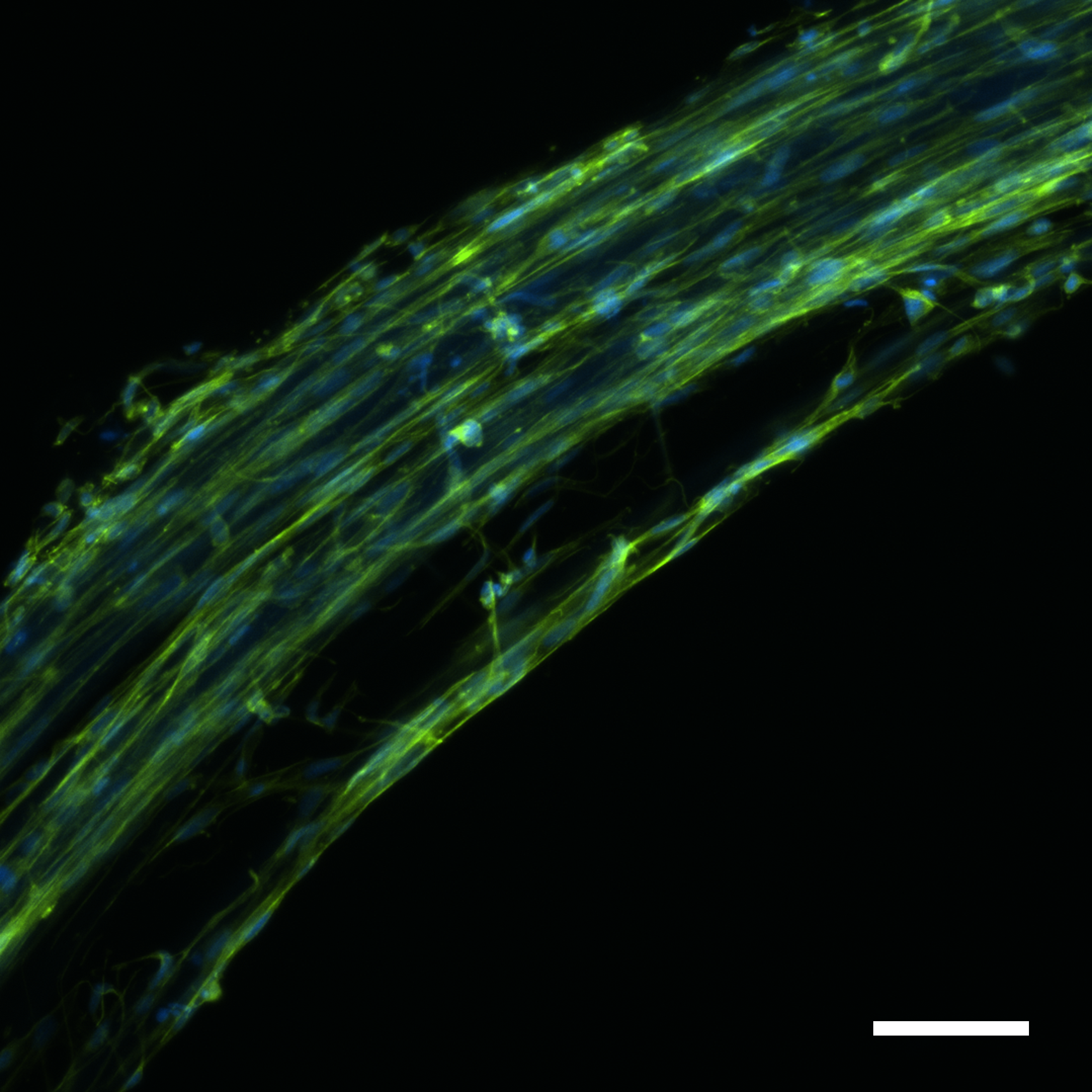

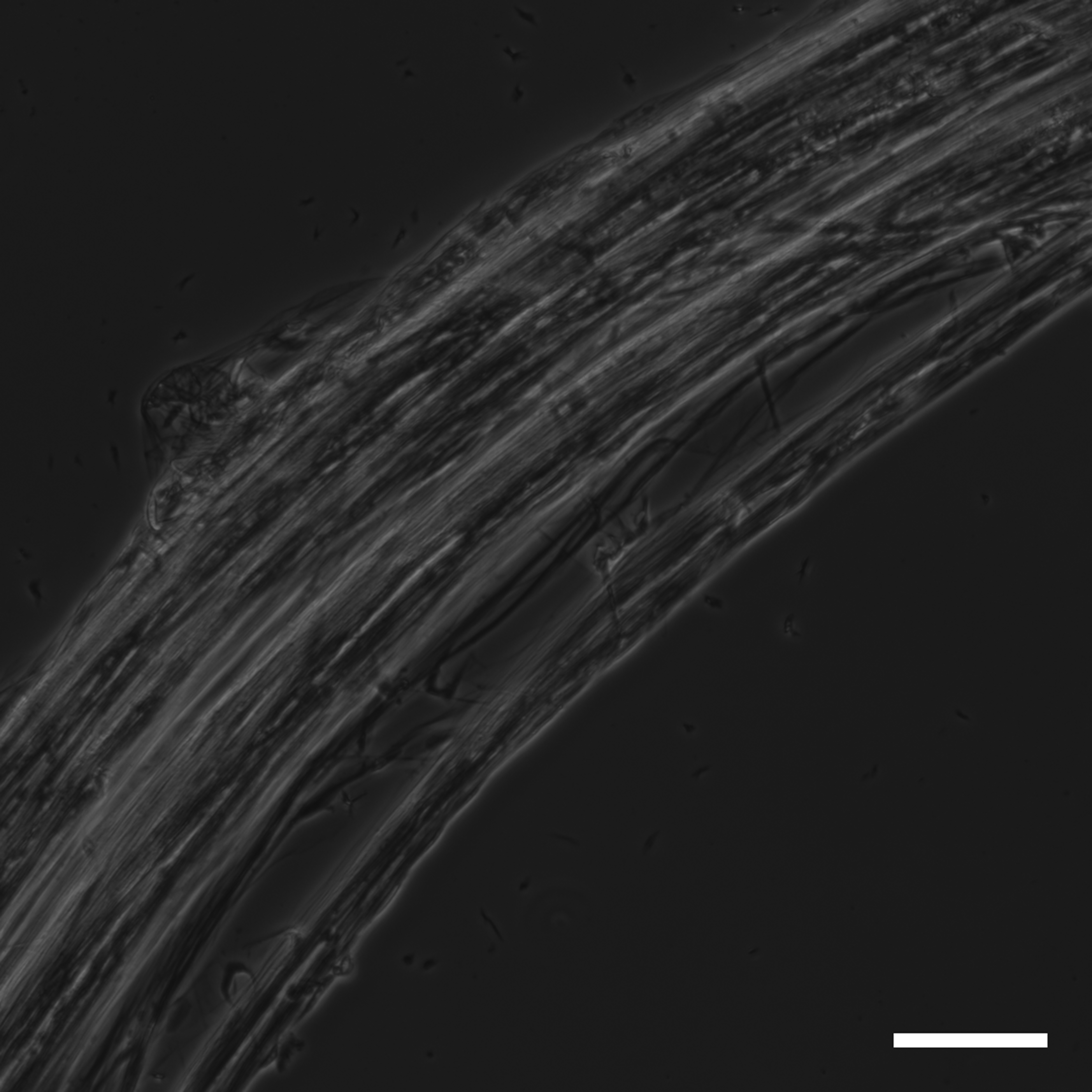


**Supplementary Figure 1. Cells integrated into a 3D network of microfibers of silk during self assembly**

MMSC integrated into (**a**) silk foam and (**b**) silk fibers, and cultured for 3 days. Left panels show f-actin (green) and nuclei (blue). Right panels show microfiber structures of silk visualized by differential interference contrast (DIC). Scale bars = 50 µm.

**
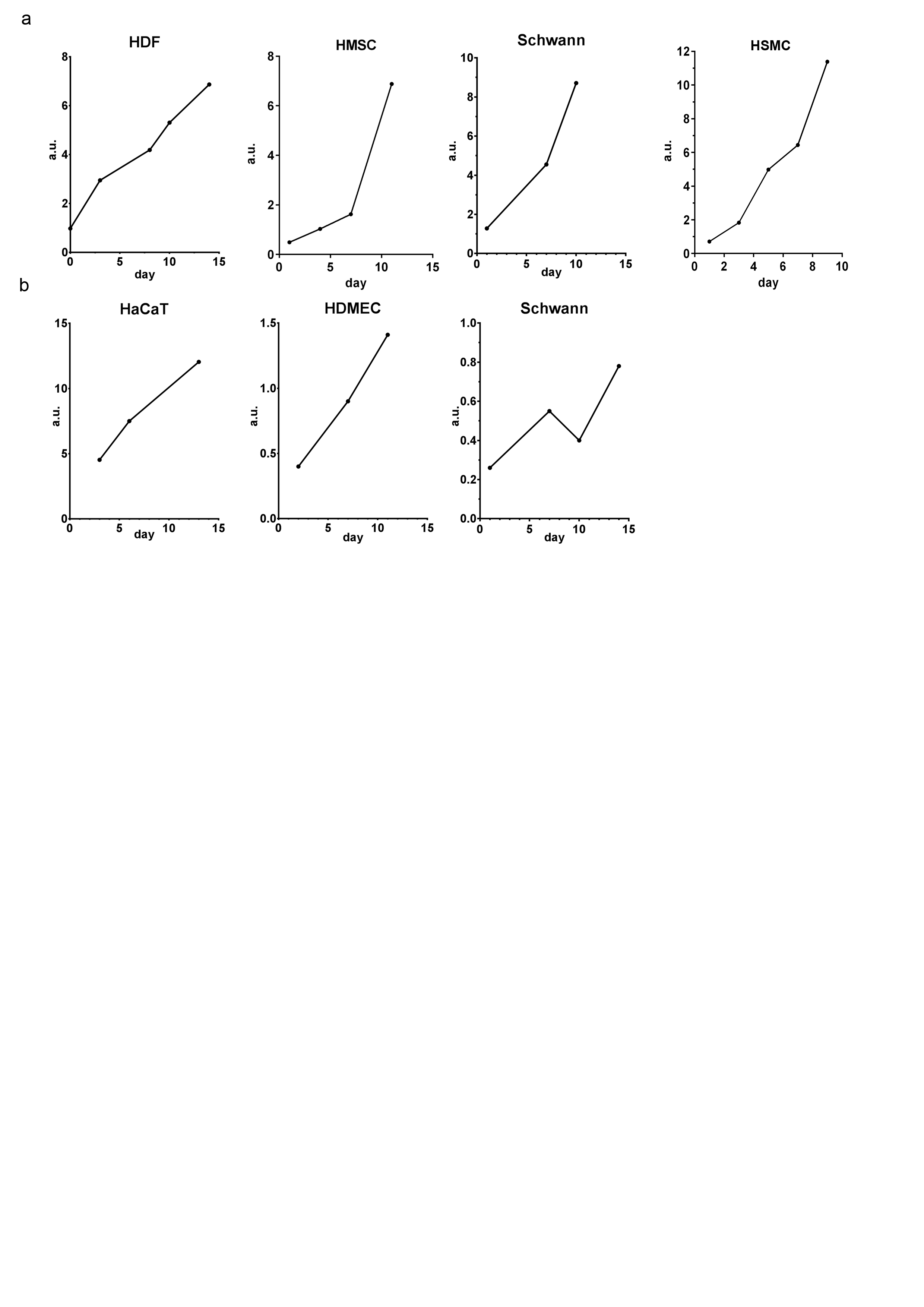
**

**Supplementary Figure 2. Cells are proliferative when integrated within FN-silk scaffolds**

Representative graphs from Alamar blue viability assay of various cell types integrated into (**a**) foam or (**b**) fiber.


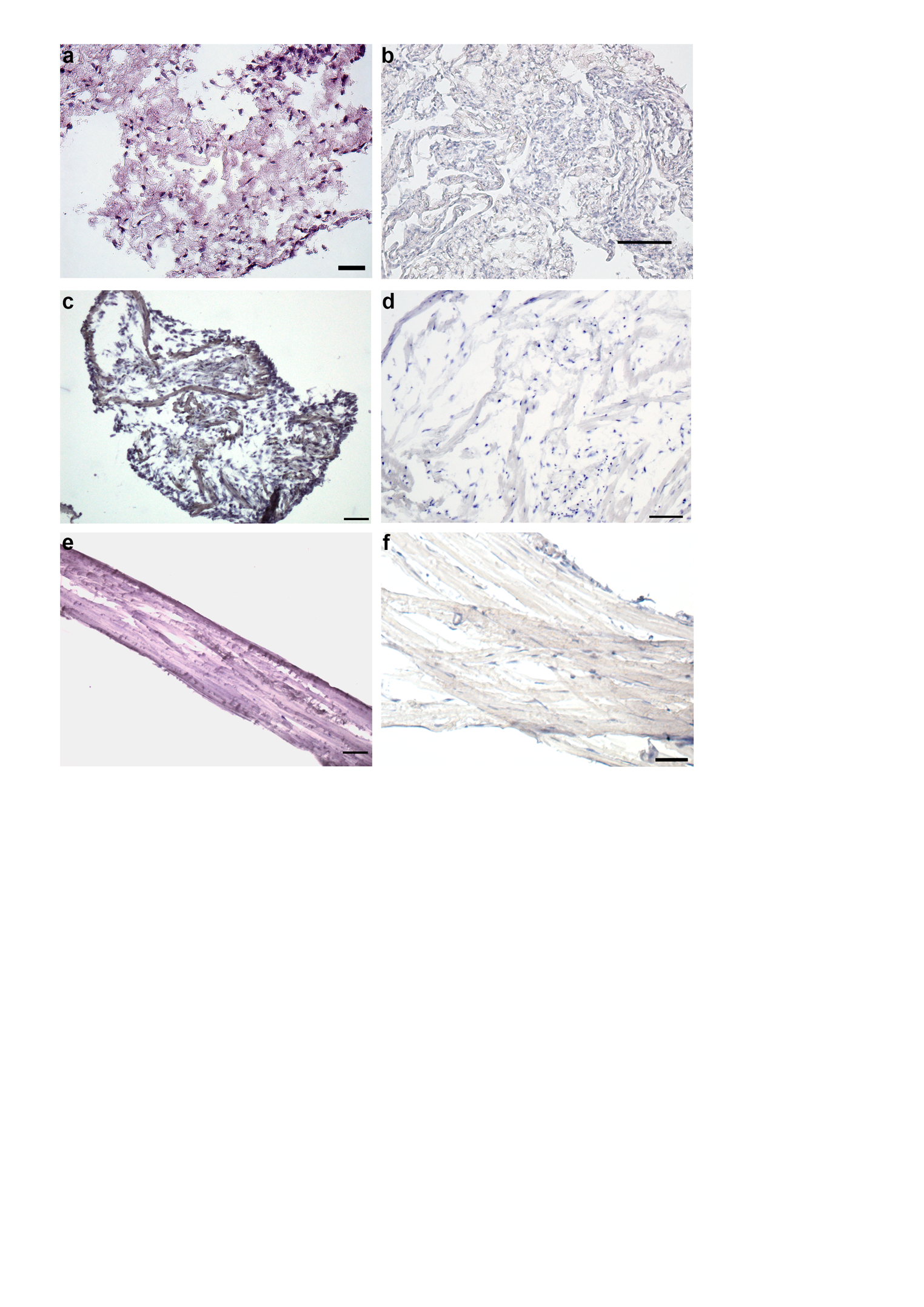


**Supplementary Figure 3. Distribution of cells throughout the 3D silk**

Hematoxylin/Eosin stained cryosections (12 µm) of FN-silk scaffolds with integrated cells. (**a**) Foam with MMSC at day 14. Scale bar = 50 µm. (**b**) Foam with endothelial cells (MDMEC) at day 14. Scale bar = 50 µm. (**c**) Cross section of fiber with endothelial cells (HDMEC) at day 16. Scale bar = 100 µm. (**d**) Cross section of fiber with fibroblasts (HDF) at day 37. Scale bar 100 = µm. (**e**) Longitudinal section of fiber with HDMEC at day 16. Scale bar = 100 µm. (**f**) Longitudinal section of fiber with HDF at day 34. Scale bar = 50 µm.


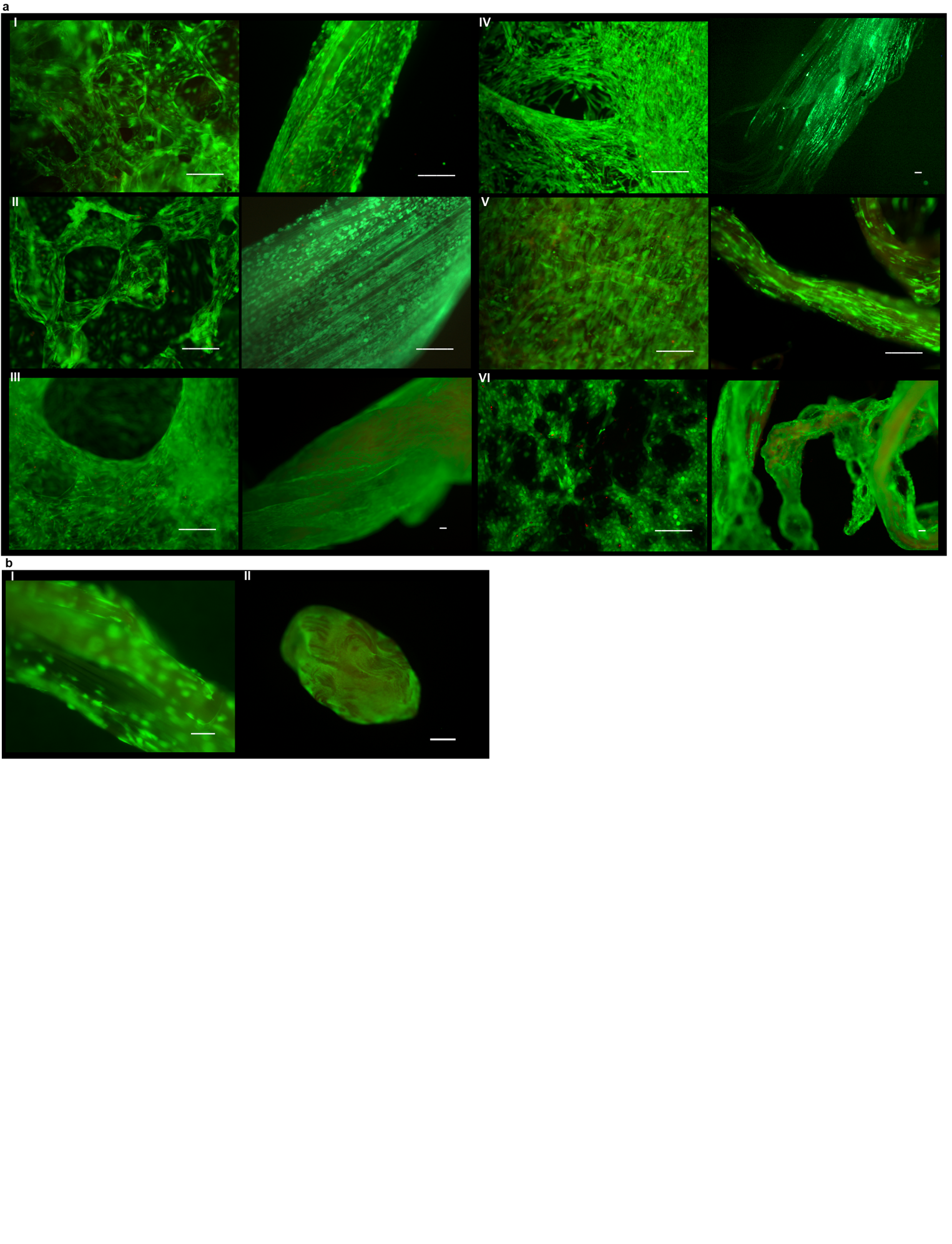


**Supplementary Figure 4. A majority of the cells within 3D silk are viable**

(**a**) Representative micrographs of live (green) and dead (bright red) stained cells integrated and cultured for 14 days in FN-silk foams (left) and fibers (right): (I) MDMEC (II) MMSC (III) HDF (IV) HMSC (V) Schwann cells and (VI) HaCaT. (**b**) Representative micrographs of live (green) and dead (bright red) stained cells in fibers integrated with (I) HDMEC, after 20 days of culture and (II) HDF, after 48 days of culture (for cell abbreviation, see Suppl. Table 1). The silk gives a dim autofluorescence in the red range.

**
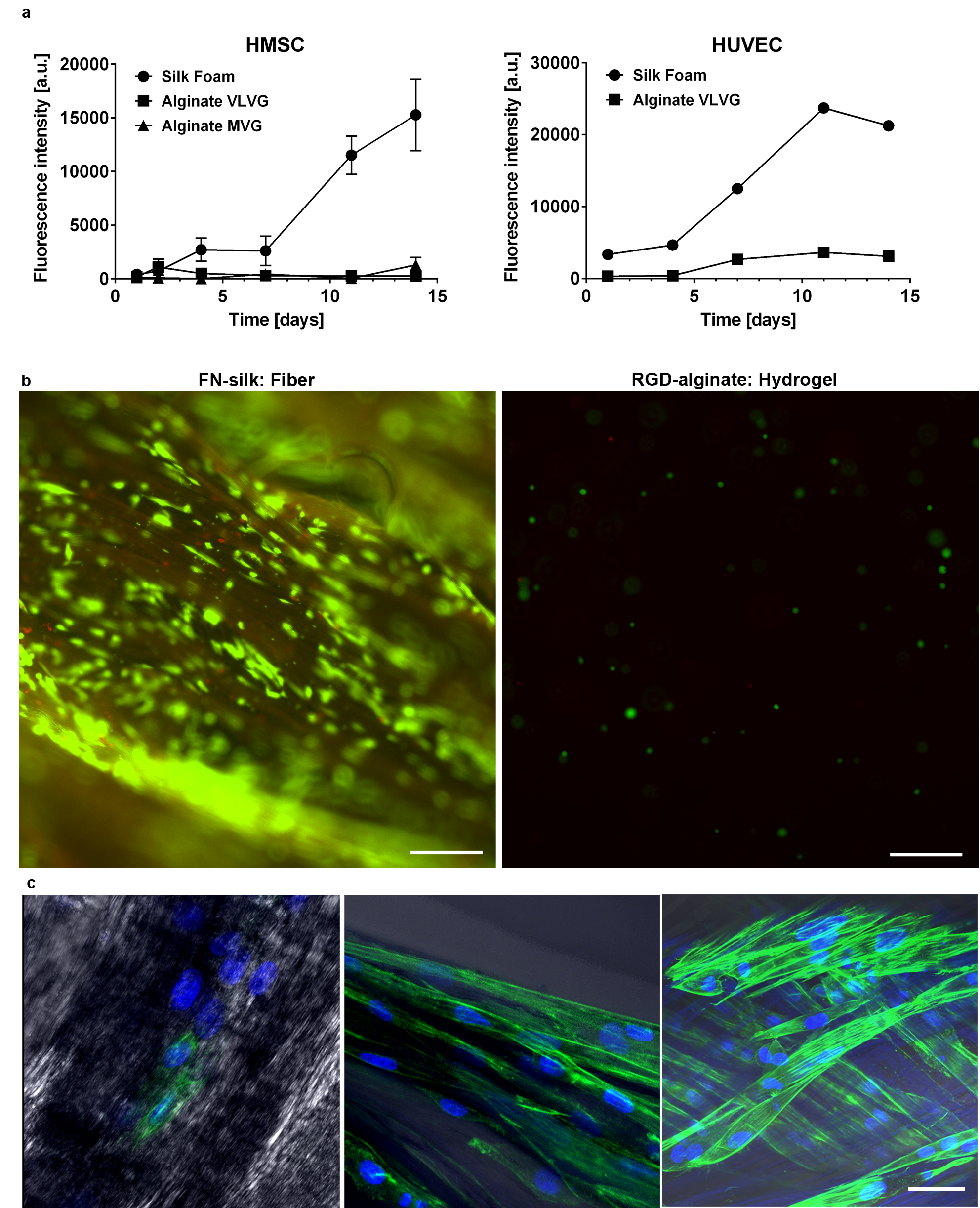
**

**Supplementary Figure 5. Viability of cells within FN-silk compared to an RGD-coupled alginate hydrogel.** (**a**) Graphs from Alamar blue viability assay of HMSC (left, N=4, n=12) and HUVEC (right, N=1, n=12) integrated into FN-silk foam (filled circle), RGD-coupled alginate hydrogel VLVG (filled square) or MVG (filled triangle). (**b**) Live (green) and dead (red) stainings of HMSC integrated into FN-silk fiber (left) and RGD-coupled alginate hydrogel MVG (right), 4 h after initial seeding (N=3, n=3). Scale bars = 200 µm. (**c**) Confocal scans of HMSCs integrated into FN-silk fiber after 1h, 4 days and 7 days (from left to right). Actin filaments are visualized by phalloidin staining (green) and cell nuclei are stained with Dapi (blue). Scale bars = 20 µm.

**
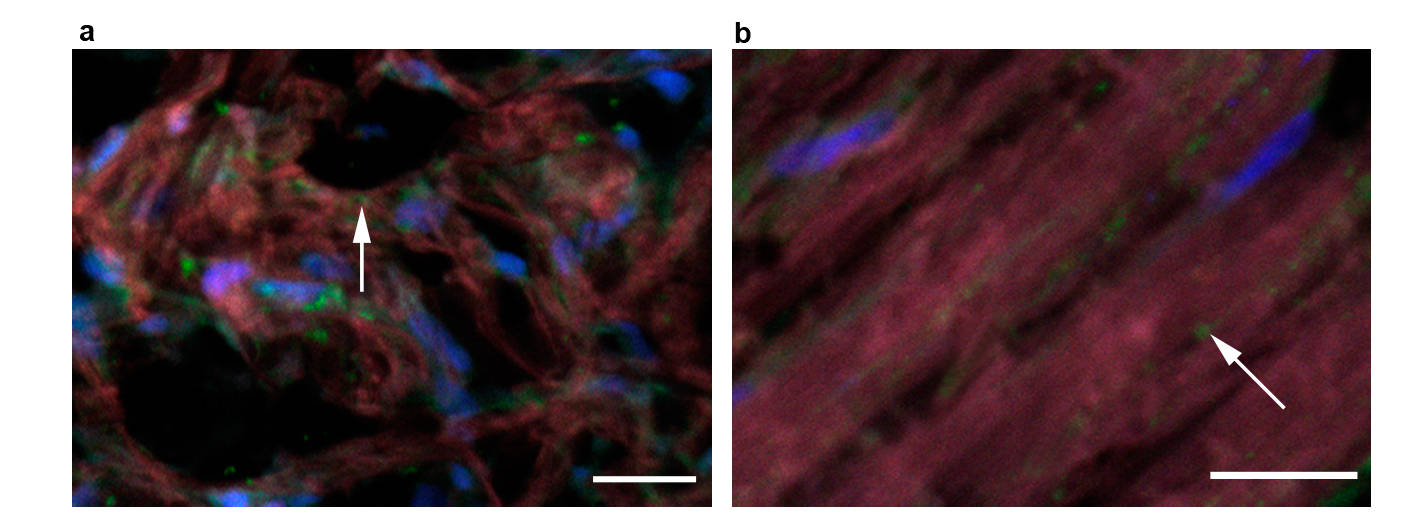
**

**Supplementary Figure 6**. **Cells within 3D silk form focal adhesions**

Focal adhesions visualized by confocal imaging after combined staining of vinculin (green, arrow mark example), F-actin (red) and nuclei (blue) of sections of (**a**) FN-silk foam with HDF at day 14 (scale bar = 30 µm) and (**b**) FN-silk fiber with HDF at day 34. Scale bars =25 µm.


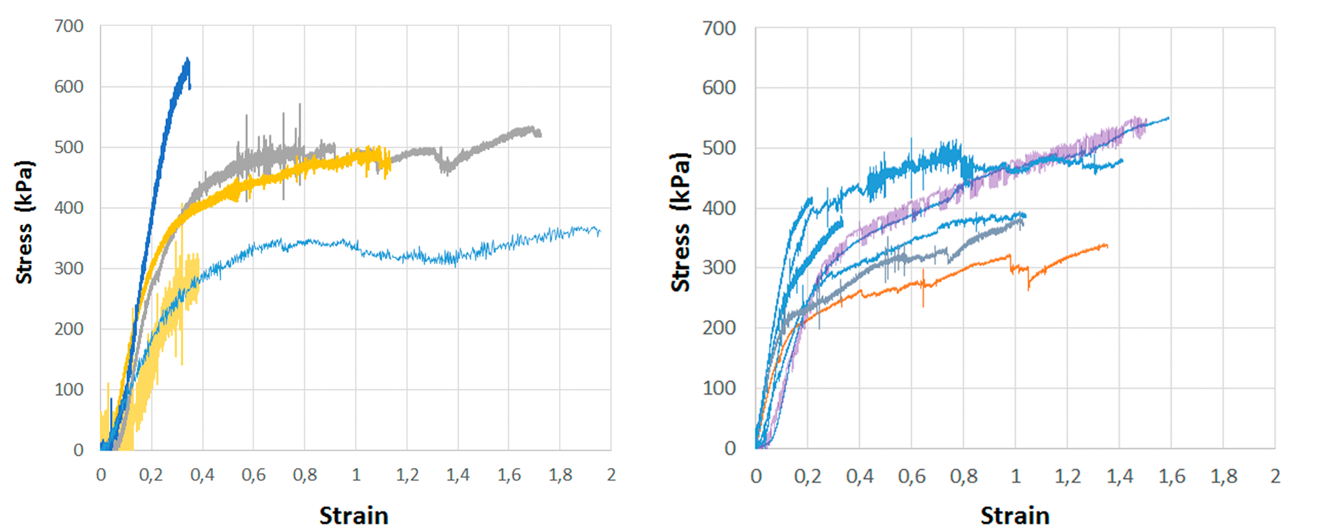


**Supplementary Figure 7. Silk fibers with integrated cells are extendable**

Stress-strain curves of two independent tensile testing experiments of FN-silk fibers with integrated mesenchymal stem cells (MMSC) after culture for 24 days (left panel) or 14 days (right panel). The tests were performed with the fibers submerged into 1xPBS buffer at 37ºC to keep the cells alive. The colors represent individual fibers.


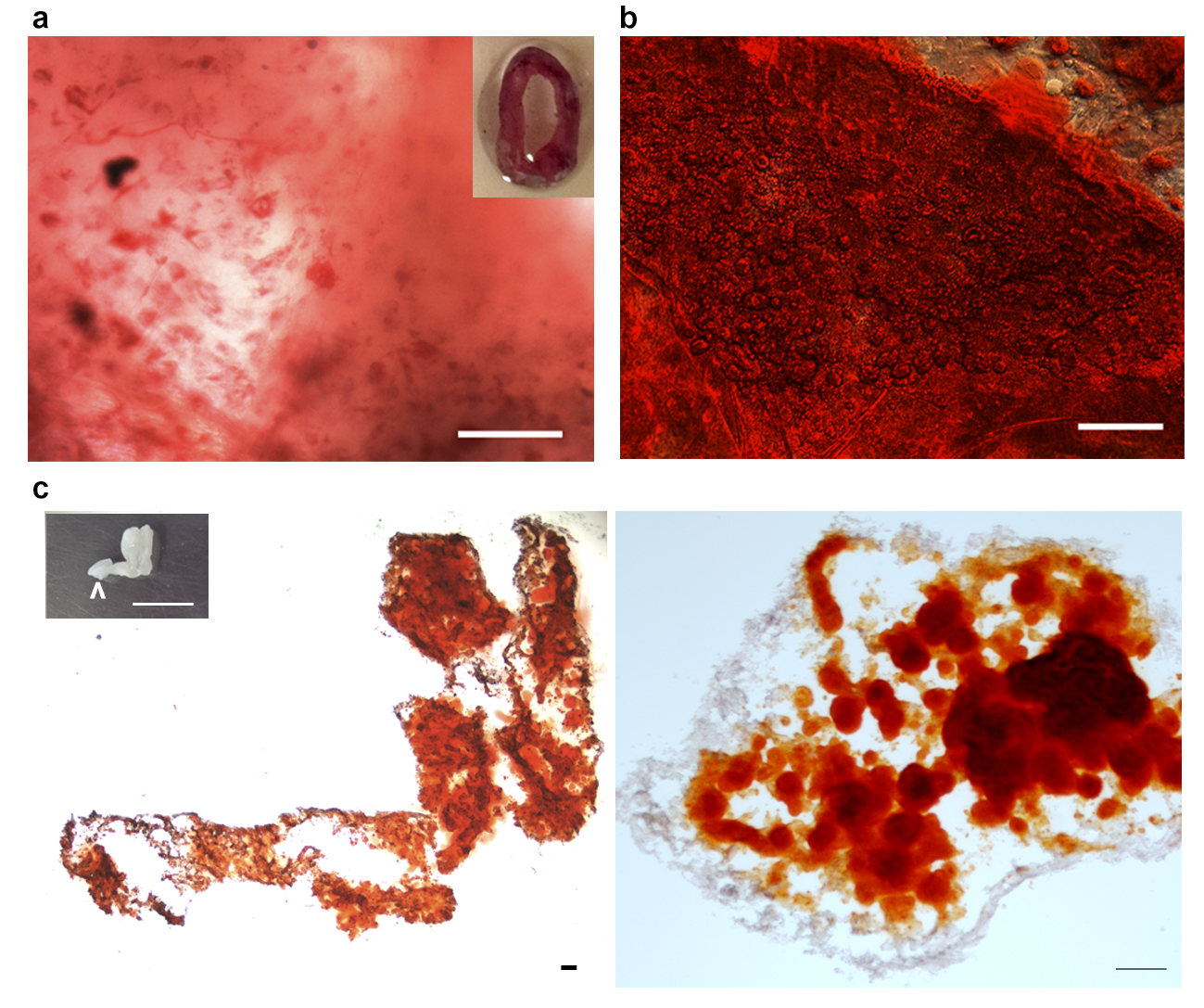


**Supplementary Figure 8. Differentiation of stem cells within 3D silk**

(**a**) Human mesenchymal stem cells (HMSC) differentiated into the adipogenic linage containing lipids, visualized by Red Oil staining (red) in FN-silk fiber, (N=2, n=4). Scale bars = 100 µm. Inset shows photo of a whole Red oil stained fiber (right), scale bar = 1 mm. (**b**) HMSCs differentiated into the osteogenic linage, probed with osteogenic marker for calcium content (Alizarin Red S (red)) in FN-silk foam. Scale bar = 100 µm) (N=2, n=4). (**c**) Cross sections of a fiber with HMSCs differentiated for 21 days into the osteogenic linage and probed with osteogenic marker for calcium content (Alizarin Red S (red)). Close up of the section to the right. Scale bars = 100 µm. Location of the close-up section is indicated with white arrow in inset (left, Scale bar = 5 mm).

**
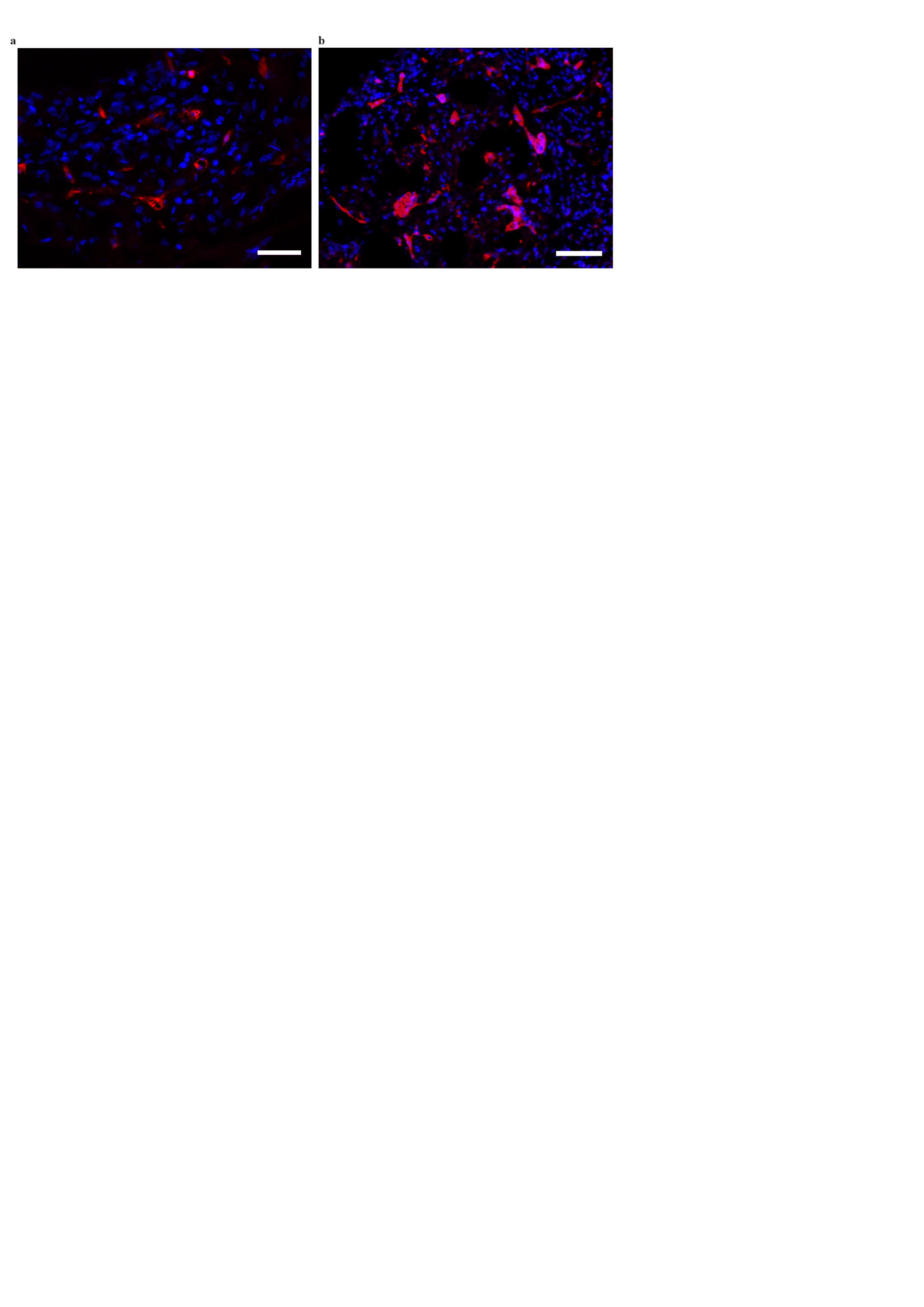
**

**Supplementary Figure 9. Vascularization within 3D silk**

Incorporation of a fraction of endothelial cells (HDMEC) together with (**a**) fibroblasts (HDF) or (**b**) skeletal muscle cells (HSkMSC) during formation of FN-silk fibers resulted in rearrangement into vessel-like structures during 11-14 days of culture. Representative cryosections stained for CD31 (red) and nuclei (blue). (N=1-8, n=1-2). Scale bars = 50 µm.

**Supplementary tables**

**Supplementary Table 1:** Cell abbreviations and full names

| **Abbreviation** | **Description** | **Referred to as:** | **Used at passage** |
| --- | --- | --- | --- |
|  |  |  |  |
| *Primary cells* |  |  |  |
| HDF | human dermal fibroblasts | fibroblasts | P8-11 |
| HDMEC | human dermal microvascular endothelial cells | human endothelial cells | P3-9 |
| HMSC | human mesenchymal stem cells | human mesenchymal stem cells | P2-8 |
| HSkMSC | human skeletal muscle satellite cells | human skeletal muscle cells | P2-6 |
| HSMC | human smooth muscle cells | human smooth muscle cells | P7 |
| HUVEC | human umbilical vein endothelial cells | HUVEC | P5 |
| MDMEC | mouse dermal microvascular endothelial cells | mouse endothelial cells | P7-9 |
| MMSC | mouse mesenchymal stem cells | mouse mesenchymal stem cells | P8-14 |
| Schwann | human Schwann cells | human Schwann cells | P2-6 |
|  |  |  |  |
| *Cell lines* |  |  |  |
| HaCaT | human keratinocyte cell line | human keratinocytes | P39-45 |
| hESC | human embryonic stem cells (HS975) | embryonic stem cells | P<25 |
| MIN6m9 | insulin-secreting mouse pancreatic β-cell line | mouse β cells | P27-35 |
|  |  |  |  |
|  |  |  |  |

| **Supplementary Table 2: Experimental repetitions and parallel replicates** | | | | | |
| --- | --- | --- | --- | --- | --- |
| **Analysis** | | **3D silk**  **format** | **Cell type** | **Number of experimental repetitions (N)** | **Number of parallel replicates (n)** |
|  |  |  |  |  |  |
| **Proliferation & Viability** | |  |  |  |  |
|  | *Alamar Blue Viability assay* | Foam | HaCaT | 3 | 4;5;6 |
|  |  |  | HDF | 5 | 2;3;9;9;9 |
|  |  |  | MDMEC | 2 | 2 |
|  |  |  | MIN6m9 | 1 | 2 |
|  |  |  | MMSC | 1 | 4 |
|  |  |  | Schwann | 2 | 3 |
|  |  |  | HUVEC | 2 | 12 |
|  |  |  | HMSC | 4 | 12 |
|  |  |  | HSMC | 1 | 7 |
|  |  |  |  |  |  |
|  |  | Fiber | HaCaT | 1 | 6 |
|  |  |  | HDF | 5 | 1;3;6;7;13 |
|  |  |  | HDMEC | 2 | 3 |
|  |  |  | HSkMSC | 9 | 2 |
|  |  |  | HUVEC | 1 | 6 |
|  |  |  | MDMEC | 1 | 3 |
|  |  |  | MMSC | 5 | 2 |
|  |  |  | Schwann | 1 | 2 |
|  |  |  |  |  |  |
|  |  | Hydrogel* | HUVEC | 2 | 9 |
|  |  |  | HMSC | 4 | 9 |
|  |  |  | HDF | 3 | 9 |
|  |  |  |  |  |  |
|  | *BrdU incorporation* | Fiber | HDF | 1 | 3 |
|  |  |  |  |  |  |
|  | *Live Dead Viability assay* | Foam | HaCaT | 3 | 2;4;7 |
|  |  |  | HDF | 2 | 2 |
|  |  |  | HMSC | 3 | 2 |
|  |  |  | MDMEC | 3 | 2 |
|  |  |  | MIN6m9 | 1 | 2 |
|  |  |  | MMSC | 3 | 2 |
|  |  |  | Schwann | 2 | 2 |
|  |  |  |  |  |  |
|  |  | Fiber | HaCaT | 3 | 2;4;7 |
|  |  |  | HDF | 5 | 1;1;2;2;3 |
|  |  |  | HMCS | 3 | 2 |
|  |  |  | HDMEC | 2 | 2 |
|  |  |  | HSkMSC | 9 | 2 |
|  |  |  | MDMEC | 3 | 2 |
|  |  |  | MMSC | 3 | 2 |
|  |  |  | Schwann | 2 | 2 |
|  |  |  |  |  |  |
|  |  | Hydrogel* | HMSC | 3 | 3 |
|  |  |  |  |  |  |
|  |  |  |  |  |  |

| **Mechanical test** | |  |  |  |  |
| --- | --- | --- | --- | --- | --- |
|  | *Tensile test* | Fiber | MMSC | 2 | 5;8 |
|  |  |  |  |  |  |
|  |  |  |  |  |  |
| **Differentiation experiments** | |  |  |  |  |
|  | *Adipogenic differentiation* | Foam | HMSC | 2 | 4 |
|  |  | Fiber | HMSC | 2 | 4 |
|  |  |  |  |  |  |
|  | *Osteogenic differentiation* | Foam | HMSC | 2 | 4 |
|  |  | Fiber | HMSC | 7 | 4 |
|  |  |  |  |  |  |
|  | *Endodermal differentiation* | Foam | hESC | 1 | 4 |
|  |  |  |  |  |  |
|  | *Differentiation into myotubes* | Fiber | HSkMSC | 12 | 4 |
|  |  |  |  |  |  |
| **Vessel formation experiments** | |  |  |  |  |
|  | *CD31+ vessel like structures* | Foam | HMSC+HDMEC | 5 | 2 |
|  |  |  | Pancreatic Islets (human) +HMSC +HDMEC | 5 | 2 |
|  |  | Fiber | HSkMSC+HDMEC | 11 | 2 |
|  |  |  | HDF+HDMEC | 1 | 1 |
|  |  |  |  |  |  |

*) Hydrogels were made of RGD-coupled Alginate (MVG and VLVG) as control to silk scaffolds

| **Supplementary Table 3:** Dimensions of fibers integrated with mouse mesenchymal stem cells for tensile testing | |
| --- | --- |
| **Characteristics** | **Mean +/- std** |
| L_0_ | 10.7 +/- 4 mm |
| Width | 394 +/- 166 µm |
| Cross-sectional area | 0.054 +/- 0.024 mm^2^ |
|  |  |

| ***Primary antibodies*** |  |  |  |  |  |
| --- | --- | --- | --- | --- | --- |
|  |  |  |  |  |  |
| **Reactivity** | **Host** | **Manufacturer** | **Clone/cat. no** | **Working conc/dilution** |  |
| BrdU | mouse | Molecular Probes | MoBU-1 | 4 ug/mL |  |
| desmin | rabbit | Prestige, Atlas Antibodies | HPA018803-100µL | 1:200 |  |
| human CD31 | mouse | Acris | BM4047 | 1:200 |  |
| human CD44 | rabbit | Abcam | ab97478 | 1:100 |  |
| SOX17 | goat | R&D | AF1924 | 1:50 |  |
| FOX2A | rabbit | Abcam | Ab40874 | 1:1000 |  |
| human vinculin | mouse | Sigma | V9131 | 9.5 ug/ml |  |
| Isotype control ab IgG1 | mouse | Sigma | MOPC-21 | Assay dependent |  |
|  |  |  |  |  |  |
| ***Secondary antibodies*** |  |  |  |  |  |
|  |  |  |  |  |  |
| **Reactivity** | **Host** | **Manufacturer** | **Thermo Fisher Cat. no** | **Dilution** | **Fluorophore** |
| mouse IgG(H+L) | goat | Molecular probes | A11034 | 1:500 or 1:1000 | AlexaFlour488 |
| mouse IgG (H+L) | goat | Molecular probes | A11003 | 1:500 or 1:1000 | AlexaFlour546 |
| rabbit IgG (H+L) | goat | Molecular probes | A11034 | 1:500 or 1:1000 | AlexaFlour488 |
| guinea pig IgG (H+L) | goat | Molecular probes | A11073 | 1:500 or 1:1000 | AlexaFlour488 |
| rabbit IgG (H+L) | donkey | Abcam | Ab150075 | 1:1000 | AlexaFlour647 |
| goat IgG (H+L) | donkey | Jackson ImmunoResearch | 705545147 | 1:1000 | AlexaFlour488 |
| rabbit IgG (H+L) | goat | Molecular probes | A11010 | 1:500 or 1:1000 | AlexaFlour546 |
|  |  |  |  |  |  |

# Supplementary Table 4: Antibody details
